# Fungal microbiota diversity in *Aedes*, *Anopheles* and *Culex* and their potential use as biological tools to control vector-borne diseases, a systematic review.

**DOI:** 10.12688/wellcomeopenres.25128.1

**Published:** 2026-02-23

**Authors:** Aicha Fatimata Sodré, Doubé Lucien Lamy, Nouhoun Traore, Abdoulaye Diabaté, Fernand Sankara, Etienne Bilgo

**Affiliations:** 1 Université Nazi Boni (UNB), Bobo-Dioulasso, 01 BP 1091, Burkina Faso; 2 Institut National de Santé Publique (INSP) / Centre Muraz, Bobo-Dioulasso, Burkina Faso; 3 Institut de Recherche en Sciences de la Santé, Direction Régionale de l’Ouest (IRSS-DRO), Bobo-Dioulasso, 01 BP 545, Burkina Faso; 4 Centre d'Excellence Africain en Innovations Biotechnologiques pour l'Elimination des Maladies à Transmission Vectorielle (CEA/ITECH-MTV), Université Nazi Boni (UNB), Bobo-Dioulasso, Burkina Faso; 5 Laboratoire des Bioressources, Agro système et Santé de l’Environnement (LaBASE), Université Nazi Boni (UNB), Bobo-Dioulasso, 01 BP 1091, Burkina Faso; 6 Unité de Formation et de Recherche en Sciences de la Vie et de la terre, Université Nazi Boni (UNB), Bobo-Dioulasso, 01 BP 1091, Burkina Faso

**Keywords:** Anopheles spp, Aedes spp, Culex spp, Fungal Microbiota, Vector control

## Abstract

**Background:**

Mosquitoes harbor diverse and dynamic microbiota which plays a critical role in shaping their development, survival and vector competence. Many studies have focused on the fungal microbiota of mosquitoes to develop new mosquito control strategies and pathogen blocking tools. This review aims to synthesize current knowledge on the composition and functional roles of the mosquito fungal microbiota, highlighting its potential as a tool for innovative vector born disease control strategies.

**Methods:**

This review was written in accordance with PRISMA guidelines. Studies on the fungal microbiota of species of the genera *Aedes*, *Anopheles*, and *Culex* and their potential in vector control were searched for in two databases (PubMed, Google Scholar). The quality of the included studies was assessed using the Joanna Briggs Institute (JBI) critical appraisal tool. Fungal diversity was investigated according to geographical distribution, mosquito species, identification techniques, developmental stages, and digestive and reproductive organs. We are interested in the antivectorial potential of fungal species.

**Results:**

A total of 32 studies were included in this review after screening 7,458 studies identified through search engines. Most studies on the fungal microbiota of species of the genera *Aedes*, *Anopheles*, and *Culex* were conducted in the United States, Brazil, European countries (France, Italy), and Africa (Kenya). Several fungal genera were isolated in the microbiota, with a predominance in species of the genus *Aedes*, followed by *Culex* and *Anopheles*. The main identification technique used was molecular biology, sometimes combined with culture characteristics. Fungal species were isolated from digestive organs (intestines, intestinal diverticulum, crop), male and female genital organs, and whole specimens at the larval and adult stages. Three phyla ( *Ascomycota*, *Basidiomycota*, and *Microsporidiomycota*) were predominant, with yeast-like, filamentous, and intracellular fungal species. Certain fungal strains inhibited the development of larval stages, while other fungal strains ( *Wickerhamomyces anomalus*, *Microsporidia* MB) reduced the vector capacity of *An. stephensi* and *An. arabiensis* species, respectively.

**Conclusion:**

Mosquitoes harbor diverse fungal microbiota in their organs with antivectorial potential which remain largely unexplored to date. Future research should explore these interactions in greater depth, particularly in organs that have been little studied, such as the salivary glands and stomach, and in regions most affected by vector-borne diseases, such as Africa. These knowledges will enable their use in vector control.

## Plain Language Summary

Mosquitoes are known to transmit many diseases such as malaria, dengue, and chikungunya. In absence of effective vaccines and growing concern of resistance to insecticides, researchers are exploring new avenues to limit transmission. One of these involves studying the fungi that live naturally in mosquito bodies to see if they could be used as allies in the fight against these vectors. This study is based on a systematic review. We analyzed 32 scientific articles that met strict selection criteria. Interesting fungi were found at different stages of the mosquito development and in different organs. Certain species were associated with pathogens blocking ability whereas other species slow down larval development and reduce mosquito survival.

## Introduction

The emergence or re-emergence of mosquito-borne diseases is sometimes linked to factors such as the evolution of pathogens, resistances developed by insect vectors, socio-demographic changes (population growth, human mobility, uncontrolled and often rapid urbanization) and climate change ( Garrido *et al.*, 2023; Zheng *et al.*, 2023). Vector-borne diseases, particularly those transmitted by mosquitoes of the *Culicidae* family, represent a major public health problem in the world ( de Bitencourt *et al.*, 2021; WHO, 2020). These diseases constitute a heavy burden for populations living especially in tropical and subtropical regions ( Katak *et al.*, 2021; Shaw & Catteruccia, 2019), and as well as a handicap to the socio-economic development of endemic countries ( Saldaña *et al.*, 2017; Shaw & Catteruccia, 2019). Mosquitoes of *Culicidae* family are widely distributed around the world and the most problematic of these belong to the genus *Aedes, Anopheles* and *Culex* ( Gao *et al.*, 2020; Rodgers *et al.*, 2017). Females mosquito vectors after mating necessarily need a blood meal as a source of protein for their eggs maturation ( LaReau *et al.*, 2023; Sarma *et al.*, 2022). When toking this blood meal biting infected human or animal hosts, female mosquitoes ingests at the same time potential pathogens ( Batson *et al.*, 2021; Fontenille *et al.*, 2017). These pathogens are transported into the insect intestines where they infect intestinal epithelial cells, then penetrate into hemolymph and invade the salivary glands, to finally being able to be transmitted to a new host during the next blood-feeding ( Duvallet *et al.*, 2018; Romoli & Gendrin, 2018). Among these pathogens transmitted by mosquitoes malaria protozoa, lymphatic filariasis ( Malassigné *et al.*, 2020; WHO, 2022) an arboviruses including the one of dengue fever, yellow fever, chikungunya, Japanese encephalitis and West Nile fever ( Gao *et al.*, 2020; Malassigné *et al.*, 2020). Bacteria such as *Rickettsia felis* responsible ( Dieme *et al.*, 2015) and *Francisella tularensis* responsible of tularemia transmitted by *Aedes* mosquitoes ( Jonckers Nieboer *et al.*, 2023), and fungi such as *Candida parapsilosis* probably responsible of candidemia and transmitted by *Anopheles*, *Aedes* and *Culex* mosquitoes ( Bozic *et al.*, 2017). The vector control constitutes a key component of combating vector-borne diseases. Insecticides were for long time the principal component of this strategy ( WHO, 2022). Unfortunately, the excessive use of insecticides agents raised chemical pollution of the environment and insect resistance ti several molecules ( Batson *et al.*, 2021; Chala & Hamde, 2021; Dahmana & Mediannikov, 2020). Innovative, complementary, and effective strategies respectful of environmental health are needed for effective vector control. The diversity and dynamics of microorganisms in mosquitoes including bacteria, fungi, viruses could constitute a base of this innovation needed. They colonize the intestine, the salivary glands, the reproductive organs and could be used for blocking the pathogen development inside the mosquitoes. For example, microorganisms such as *Asaia* bacteria ( Favia *et al.*, 2007), *Wolbachia* ( Bian *et al.*, 2013), *Pantoea stawartii* et *Pantoea agglomerans* ( Lindh *et al.*, 2008; Wang *et al.*, 2012), *Serratia marcescens* ( Bando *et al.*, 2013; Wang *et al.*, 2017), entomopahogenic fungi ( *Metarhizium anisopliae*) ( Fang *et al.*, 2011) and fungal microbiota ( *Wickerhamomyces anomalus*) ( Cappelli *et al.*, 2021) have been proposed for malaria control.

Entomopathogenic fungi (such as *Beauveria*, *Metarhizium*) have proven to be effective biological control agents due to their efficacy and lack of impact on non-target organisms ( Perumal *et al.*, 2024). In Burkina Faso, the virulence of local strains of *Metarhizium pingshaense* (Met_S10; Met_S26) has been tested against *Anopheles coluzzii* mosquitoes ( Bilgo *et al.*, 2018). In addition, when combined with a chemical insecticide (Deltamethrin), these different local strains (Met_S10; Met_S26, Met_S31) made the mosquitoes more susceptible to death by the chemical ( Lamy *et al.*, 2025).

Several studies have focused on the diversity of the fungal microbiota of mosquitoes species of genera *Aedes*, *Anopheles* and *Culex* ( Flores *et al.*, 2022; Hegde *et al.*, 2024; Nattoh *et al.*, 2021a) and the interest of certain fungi in the symbiotic fight against malaria ( Angleró-Rodríguez *et al.*, 2016; Cappelli *et al.*, 2019; Herren *et al.*, 2020; Valzano *et al.*, 2016) and certain arbovirosis ( Angleró-Rodríguez *et al.*, 2017)

This systematic review is carried out to synthesize current knowledge on the fungal microbiota diversity in mosquito genera *Aedes*, *Anopheles* and *Culex* and analyze the perspectives for using them as biological control tools against vector-borne diseases.

## Methods

### Data collection

This review was written in accordance with the PRISMA (Preferred Reporting Items for Systematic Reviews and Meta-Analyses) (2020) protocol for systematic reviews ( Page *et al.*, 2021). A duly completed PRISMA checklist was attached (Supplementary File 1. Table I).

Research papers used in this study were collected in two electronic databases from September 2023 to April 3, 2025 including PubMed and Google Scholar. From the keywords *Anopheles* spp, *Aedes* spp *, Culex* spp, fungi microbiota and vector control we built a research algorithm using Boolean operators as follow (( *Anopheles* spp) AND ( *Aedes* spp) AND ( *Culex* spp) AND (Fungi microbiota) AND (Vector control) OR ( *Anopheles* spp) OR ( *Aedes* spp) OR ( *Culex* spp) OR (Fungi microbiota) OR (Vector control)).

The publication date filter for this review, set at 20 years, aims to provide an up-to-date and in-depth analysis of the fungal communities of mosquitoes of genera *Aedes*, *Anopheles* and *Culex* and their potential in vector control. We also conducted a manual search of the reference lists of identified studies and previously published reviews to identify other relevant studies. No articles from sources were added in order to preserve the systematic nature of this analysis.

**Figure 1.**
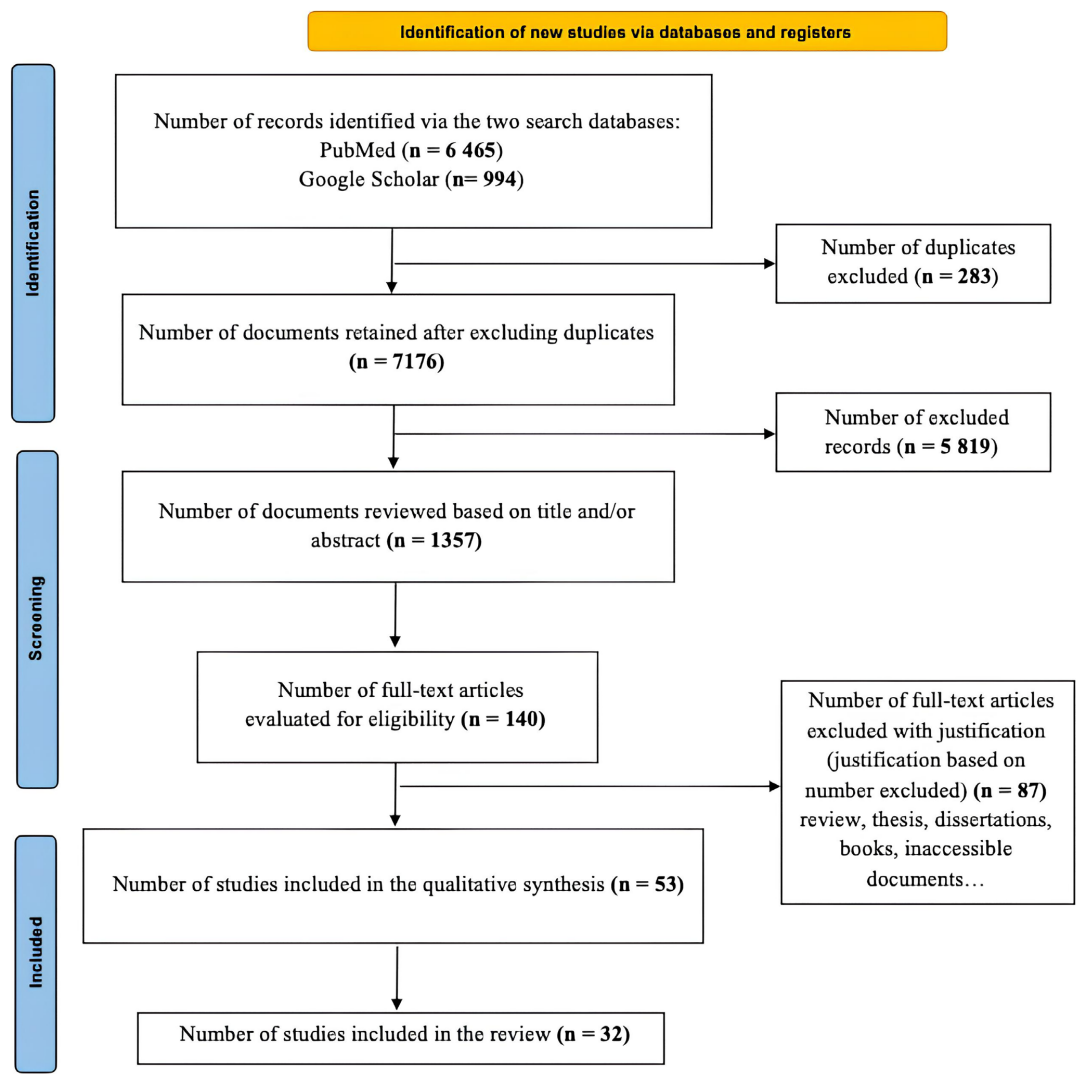
PRISMA flow diagram showing the study selection process.

The article databases were first imported into Rayyan software for manual filtering ( https://www.rayyan.ai/ consult on April 3, 2025 ( Ouzzani *et al.*, 2016). Using this software, we first eliminated duplicate articles, then eliminated irrelevant articles and sorted the articles to be included according to the inclusion criteria. Rayyan software enabled the development of the PRISMA diagram ( Figure 1).

### Eligibility criteria

**Inclusion criteria:**

- Studies published in the last 20 years (2005–2025)
- Studies published in english in pure reviewed journal
- Studies published on any of the target mosquito genera ( *Aedes*, *Anopheles* and *Culex*)
- Studies tolking about fungal and vector control

**Exclusion criteria**:

- All studies not fiting with the inclusion criteria listed
- Review studies
- Dissertations and theses
- Inaccessible articles

### Eligibility assessment and data extraction

The articles were sorted manually and independently by four authors. These authors first reviewed the titles and abstracts and discussed any discrepancies until a consensus was reached. Next, each author independently reviewed the titles and abstracts of all extracted articles. In cases of disagreement, consensus was reached by reviewing the full text of the various articles. If necessary, a fifth and sixth author were consulted for the final decision. Four authors then independently reviewed the full-text articles for inclusion. However, in the event of further disagreements, consensus was reached through discussion of the inclusion or exclusion criteria. Otherwise, the fifth and sixth authors were consulted.

Discrepancies were resolved by comparing the extracted data. The authors verified the accuracy of the extracted data.

### Quality and risks of bias evaluation of included the studies

The quality and risks of bias of included studies was determined using the Joanna Briggs Institute (JBI) critical appraisal tool, which comprises 9 parameters ( https://jbi.global/critical-appraisal-tools) to cohort, case-control, or cross-sectional studies.

A score of “1” was assigned to each parameter that was well completed and “0” if it was not described. The top-quality score was 9. The quality of each study was assessed as high if it was ≥ 7 points, moderate if the score was between 4 and 6 points, and low if the score was < 4 points. The quality of the studies was assessed independently by three evaluators and a fourth evaluator for consensus in case of disagreement.

### Data summary

The following variables were collected: lead author, study location, mosquito origin (field or laboratory), developmental stage (larvae, adults), isolation and identification techniques, dissected tissues (whole larvae, whole adults, organs), mosquito vector species of the genera *Aedes*, *Anopheles*, and *Culex*, and finally, if available, the potential antivector properties of fungal species in the mosquito microbiota.

A map of the geographical distribution of the studies was developed using information on the location where the mosquitoes were collected or just the country of study when articles did not provide the sample origin. This map was created using QCIS 3.4 software. In addition, Microsoft Excel was used to create graphs providing information on fungal diversity according to species of the genera *Aedes*, *Anopheles*, and *Culex*, as well as according to the isolation and identification techniques used in each study included.

## Results

### Study results

Of the total of 7,458 articles initially were identified, only thirty-two (32) fully met the eligibility criteria after removing duplicates (283) and applying the inclusion and exclusion criteria. Three (3) articles discussing the antivectorial potential of two fungal strains appeared to meet the inclusion criteria but were excluded because the strains came from previous studies that did not meet the selection criteria ( Barnard *et al.*, 2007; Frankel-Bricker, 2020; Frankel-Bricker *et al.*, 2020).

There were 12 studies associating the diversity of microorganisms (fungi and/or bacteria, viruses) in the microbiota of mosquito species. More specifically, only four included studies focused on the anti-plasmodial potential of certain fungal strains ( Cappelli *et al.*, 2014; Cappelli *et al.*, 2019; Herren *et al.*, 2020; Valzano *et al.*, 2016) among articles studying the diversity of the fungal microbiota of *Anopheles* mosquitoes responsible of malaria. No study on the diversity of the fungal microbiota of mosquitoes in *Aedes* and *Culex* responsible of arboviruses infections have been investigated their antiviral potential.

### Risks of bias

The Joanna Briggs Institute (JBI) critical appraisal tool, comprising nine points, was used to assess the quality of the studies. The different thresholds of the bias risk checklist are pointed out in the Supplementary File 2. Table II. Of the 32 studies included and evaluated, 28 studies correctly met the 9 points of the checklist with a threshold of 100%, indicating a low risk rate.

With a threshold of 77.78%, two studies each presented a high risk of bias and an uncertain risk of bias due to the non-representativeness of the target population and inappropriate statistical analysis, respectively ( Gusmão *et al.*, 2007; Tajedin *et al.*, 2009). Furthermore, with a threshold of 88.89% each, two studies presented a high risk of bias due to inappropriate statistical analysis ( da S Pereira *et al.*, 2009) and an uncertain risk of bias due to a small sample size ( Chandler *et al.*, 2015), respectively.

In general, all studies from the two databases (PubMed, Google Scholar) evaluated and included in the systematic review were of high quality, thereby reinforcing knowledge about the fungal diversity of species of the genera *Aedes*, *Anopheles*, and *Culex* and their antivectorial potential.

### Study characteristics

The detailed characteristics of the studies are presented in the Supplementary file 3. Table III.

***Geographical distribution of studies on fungal diversity in the microbiota of mosquitoes of the genera Aedes, Anopheles, and Culex***

**Figure 2.**
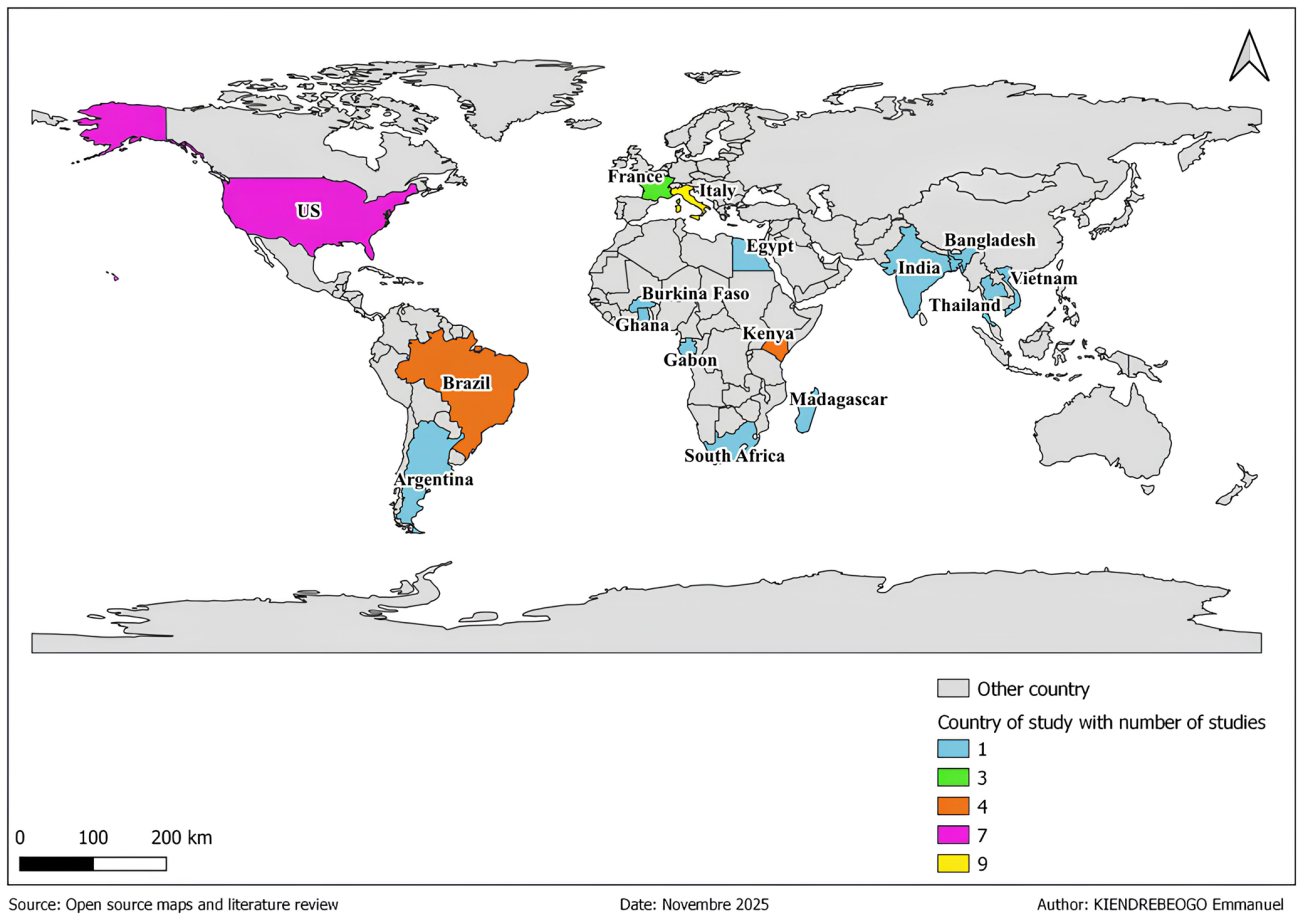
Geographic distribution of published studies investigating the fungal microbiota associated with *Aedes*, *Anopheles*, and *Culex* mosquitoes.

The map in Figure 2 shows the global distribution of studies on the fungal microbiota of *Aedes*, *Anopheles*, and *Culex* mosquitoes. Countries are represented by different colors depending on the number of studies. The number of studies per country ranges from 0 to 9.

The gray color represents countries where no studies were found according to research and inclusion criterias. These resulting countries include several countries in Central and West Africa, in Central Asia and in Oceania.

Countries with 1 to 2 studies includes in this review are represented in color light blue in the map. These countries include South Africa ( Steyn *et al.*, 2016), Argentina ( Flores *et al.*, 2022), South Asia ( Bozic *et al.*, 2017), Burkina Faso ( Bozic *et al.*, 2017), Egypt ( Galal *et al.*, 2017), Gabon ( Zouache *et al.*, 2022), Ghana ( Akorli *et al.*, 2024), India ( Tajedin *et al.*, 2009), Madagascar ( Luis *et al.*, 2019), Thailand ( Thongsripong *et al.*, 2017; Thongsripong *et al.*, 2021), and Vietnam ( Luis *et al.*, 2019).

In France, represented in light green color, three studies have focused on the fungal microbiota of *Aedes* mosquitoes ( Luis *et al.*, 2019; Guégan *et al.*, 2020a; Malassigné *et al.*, 2025).

Furthermore, in countries such as Brazil ( Gusmão *et al.*, 2007; da S Pereira *et al.*, 2009; Gusmão *et al.*, 2010; Bozic *et al.*, 2017) and Kenya ( Herren *et al.*, 2020; Nattoh *et al.*, 2021a; Onchuru *et al.*, 2024) represented in color orange, four studies focused on the fungal microbiota *Aedes* and *Anopheles* mosquitoes.

In addition, seven studies focused on the fungal microbiota of *Aedes*, *Anopheles*, and *Culex* mosquitoes in the United States, represented in color light purple ( Angleró-Rodríguez *et al.*, 2016; Angleró-Rodríguez *et al.*, 2017; Bishop-Lilly *et al.*, 2010; Chandler *et al.*, 2015; Hegde *et al.*, 2024; Muturi *et al.*, 2016; Tawidian *et al.*, 2021).

Finally, Italy, represented in color yellow on the map, is the country where several studies (9) have been conducted on the fungal microbiota of *Aedes*, *Anopheles*, and *Culex* ( Bozic *et al.*, 2017; Cappelli *et al.*, 2014; Cappelli *et al.*, 2019, Cappelli *et al.*, 2023; Ricci *et al.*, 2011a; Ricci *et al.*, 2011b; Valzano *et al.*, 2016).

There is an uneven distribution of studies, with a greater number of studies in Italy, the United States, and Brazil. The lack of data in certain countries such as Central Africa, several West African countries, Central Asia, and Oceania may lead to geographical bias in scientific writing on the fungal microbiota of *Aedes*, *Anopheles* and *Culex* mosquitoes, considering that certain fungal species may be specific ecosystem.

***Fungal diversity associated with the microbiota of *Aedes, Anopheles*, and *Culex* species***

The analyse of the distribution of fungal genera in the microbiota according to mosquito genera ( *Aedes*, *Anopheles* or *Culex*) showed variation in the fungal microbiota composition of the microbiota of certain mosquito species. These fungal genera are most often composed of yeast-like fungi and filamentous fungi that can be commensal, symbiotic, or pathogenic to mosquitoes.

**Fungal diversity associated with the microbiota of *Aedes* species**

**Figure 3.**
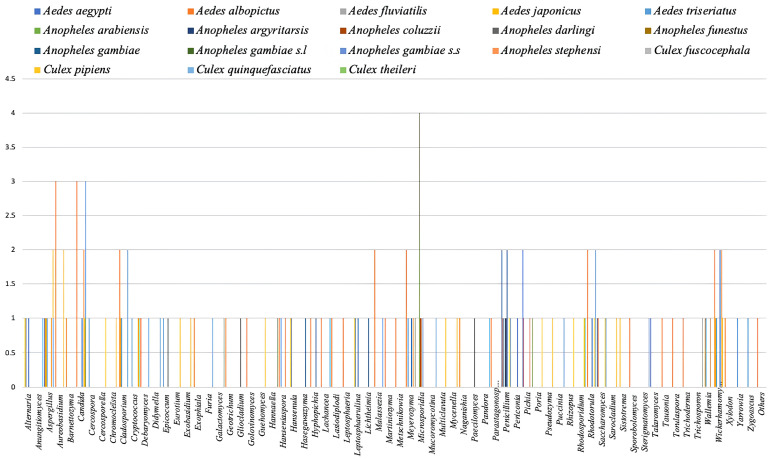
Distribution of fungal genera in the mosquito microbiota according to mosquito genera ( *Aedes*, *Anopheles* and *Culex*).

The analyse of the *Aedes* fungal microbiota according to literature showed that studies were focused on species such as *Aedes aegypti* ( *Ae. aegypti)*, *Aedes albopictus* ( *Ae. albopictus*), *Aedes fluviatilis* ( *Ae. fluviatilis*), *Aedes japonicus* ( *Ae. japonicus*), and *Aedes triseriatus* ( *Ae. triseriatus*), represented by different colors respectively ( Figure 3).

In total of eight fungal genera, including five genera belonging to yeast-like fungi and three genera belonging to filamentous fungi (molds), *Ae. aegypti* has a relatively undiversified microbiota, with a predominance of the fungi genera belonging to *Candida*, *Penicillium*, *Pichia*, and *Wickerhamomyces*. Although weakly associated with this mosquito species, yeast-like fungi such as the genera *Meyerozyma* and *Saccharomyces* ( Bishop-Lilly *et al.*, 2010; Bozic *et al.*, 2017) and molds such as the genera *Aspergillus* and T *alaromyces* ( Angleró-Rodríguez *et al.*, 2017; Bishop-Lilly *et al.*, 2010) have been identified.

Unlike *Ae. aegypti*, with more than thirty fungal genera, including 17 genera of yeast-like fungi and 11 genera of filamentous fungi (molds, phytopathogens), *Ae. albopictus* has a more diverse microbiota with a predominance of *Aureobasidium*, *Candida*, *Cladosporium*, *Malassezia*, *Meyerozyma*, *Rhodotorula*, and *Wickerhamomyces*. Yeast-like fungal genera weakly associated with *Ae. albopictus* include *Barnettozyma*, *Debaryomyces*, *Geotrichum*, *Hanseniaspora*, *Hyphopichia*, *Lachancea*, *Martiniozyma*, *Metschnikowia*, *Naganishia*, *Pichia*, *Saccharomyces*, *Sporobolomyces*, *Tausonia*, and *Torulaspora* ( Bozic *et al.*, 2017; Hegde *et al.*, 2024; Luis *et al.*, 2019; Malassigné *et al.*, 2025). As for filamentous fungi, the weakly identified genera belong to molds such as *Aspergillus*, *Exophiala*, *Penicillium*, and *Trichoderma* ( Guégan *et al.*, 2020a; Luis *et al.*, 2019; Malassigné *et al.*, 2025) phytopathogens including *Golovinomyces*, *Lasiodiplodia*, *Parastagonospora*, *Leptosphaeria*, and *Xylodon* ( Guégan *et al.*, 2020a).

Furthermore, *Ae. fluviatilis* harbors a relatively weak microbiota consisting solely of a filamentous fungus of the genus *Aspergillus* ( da S Pereira *et al.*, 2009).

Finally, the two species *Ae. japonicus* and *Ae. triseriatus* appear to harbor microbiota that are not very diverse but identical in filamentous fungi, including molds such as the genera *Alternaria*, *Aspergillus*, *Cladosporium*, and *Penicillium*, and phytopathogens such as *Cercospora*, *Epicoccum*, and *Sistotrema* ( Muturi *et al.*, 2016). The yeast-like fungi common to these two-mosquito species include the genera *Hansenula*, *Meyerozyma*, *Wallemia*, *Wickerhamomyces*, *Yarrowia*, and *Zygoascus* ( Muturi *et al.*, 2016). Specifically, the genera *Cryptococcus* and *Hanseniaspora* have been identified in the microbiota of *Aedes triseriatus* ( Muturi *et al.*, 2016).

**Fungal diversity associated with the microbiota of *Anopheles* species**

The analyse of the *Anopheles* mosquitoes fungal microbiota according to this review showed that *Anopheles* included studies were focused on the species including such as *Anopheles arabiensis* ( *An. arabiensis*), *Anopheles argyritarsis* ( *An. argyritarsis*), *Anopheles coluzzii* ( *An. coluzzii*), *Anopheles darlingi* ( *An. darlingi*), *Anopheles funestus* ( *An. funestus*), *Anopheles gambiae* ( *An. gambiae*), *Anopheles gambiae* s.l. ( *An. gambiae* s.l.), *Anopheles gambiae* s.s. ( *An. gambiae* s.s.) and *Anopheles stephensi* ( *An. stephensi*), represented respectively in different colors ( Figure 3).

Species such as *An. arabiensis*, *An. coluzzii*, *An. funestus*, *An. gambiae* s.l, and *An. gambiae* s.s. each harbor a microbiota poor fungal microbiota, with only one genus ( *Microsporidia*) and specifically intracellular ( Akorli *et al.*, 2024; Herren *et al.*, 2020; Nattoh *et al.*, 2021b; Onchuru *et al.*, 2024). This genus *Microsporidia* was predominant in *An. arabiensis*.

In addition, *An. argyritarsis* and *An. darlingi* showed a poor microbiota, with only one genus ( *Penicillium*) and three genera ( *Penicillium*, *Gliocladium*, *Paecilomyces*) respectively, all of which are filamentous fungi ( da S Pereira *et al.*, 2009).

On the other hand, with a predominance of the genus *Wickerhamomyces*, *An. gambiae* hosts a fairly diverse fungal microbiota with six genera of yeast-like fungi and seven genera of filamentous fungi. Yeast-like fungi such as the genera *Candida*, *Hasegawazyma*, *Hyphopichia*, *Meyerozyma*, and *Rhodotorula* ( Bozic *et al.*, 2017; Nattoh *et al.*, 2021a) as well as filamentous fungi including molds (genera *Alternaria*, *Penicillium*, *Periconia*) ( Angleró-Rodríguez *et al.*, 2016; Nattoh *et al.*, 2021a) and certain phytopathogens ( *Epicoccum*, *Leptosphaerulina*, *Lichtheimia*) ( Nattoh *et al.*, 2021a) have been weakly associated with *An. gambiae*.

Finally, *An. stephensi* harbors a less diverse fungal microbiota including six genera of yeast-like fungi and two genera of filamentous fungi. While the genera *Candida* and *Wickerhamomyces* are predominant, other yeast-like genera such as *Hanseniaspora*, *Meyerozyma*, *Pichia*, and *Wallemia* ( Bozic *et al.*, 2017; Ricci *et al.*, 2011a) and a few filamentous genera such as *Aspergillus* and *Penicillium* ( Angleró-Rodríguez *et al.*, 2016; Tajedin *et al.*, 2009) are weakly associated with *An. gambiae*.

**Fungal diversity associated with the microbiota of *Culex* species**

The results showed that for *Culex* mosquitoes fungal microbiota, the included studies were focused on species including such as *Culex fuscocephala* ( *Cx. fuscocephala*), *Culex pipiens* ( *Cx. pipiens*), *Culex quinquefasciatus* ( *Cx. quinquefasciatus*), and *Culex theileri* ( *Cx. theileri*), represented by different colors ( Figure 3).

*Cx. pipiens* appears to harbor a microflora rich in fungi of eight yeast-like genera (Candida, *Cryptococcus*, *Guehomyces*, *Malassezia*, *Pseudozyma*, *Rhodosporidium*, *Rhodotorula*, *Saccharomyces*, *Wickerhamomyces*), 10 filamentous genera with molds (Aspergillus, *Aureobasidium*, *Cladosporium*, *Eurotium*, *Penicillium*, *Rhizopus*) and phytopathogens ( *Cercosporella*, *Exobasidium*, *Mycenella*, *Sarocladium*) ( Chandler *et al.*, 2015; Galal *et al.*, 2017; Steyn *et al.*, 2016).

lichenized ( *Multiclavuta*) and lignivorous ( *Mycenella*, *Poria*) fungi genera have been also found ( Chandler *et al.*, 2015). All other fungal genera are found weakly associated to *Cx. Pipiens* except two predominant genera *Aspergillus* and *Aureobasidium*.

Some fungi with unspecified genera belonging to various phyla, including *Ascomycota*, *Basidiomycota*, and *Zoopagomycota*, have been identified in the *Cx. fuscocephala* microbiota ( Thongsripong *et al.*, 2021).

Furthermore, microbiota undiversified *Cx. Pipiens* and *Cx. quinquefasciatus* species even undiversified, harbors fungi from seven yeast-like genera ( *Candida*, *Debaryomyces*, *Lachancea*, *Malassezia*, *Meyerozyma*, *Rhodotorula*, and *Sterigmatomyces*) ( Bozic *et al.*, 2017; Flores *et al.*, 2022; Hegde *et al.*, 2024), eight filamentous genera with molds ( *Anungitiomyces*, *Aspergillus*, *Cladosporium*, *Mucoromycotina*, and *Penicillium*), phytopathogens ( *Didymella*, *Puccinia*), and entomopathogens ( *Furia*) ( Flores *et al.*, 2022; Thongsripong *et al.*, 2017). A predominance of *Candida*, *Cladosporium*, and *Rhodotorula* fungi was observed among the genera associated with *Cx. quinquefasciatus*.

Finally, only yeast-like fungi from nine genera ( *Candida*, *Cryptococcus*, *Galactomyces*, *Hannaella*, *Meyerozyma*, *Pichia*, *Rhodosporidium*, *Rhodotorula*, *Trichosporon*) were found in microbiota *Cx. theileri* ( Steyn *et al.*, 2016).

***Fungal diversity associated with the microbiota of Aedes, Anopheles, and Culex species according to culture techniques***

**Figure 4.**
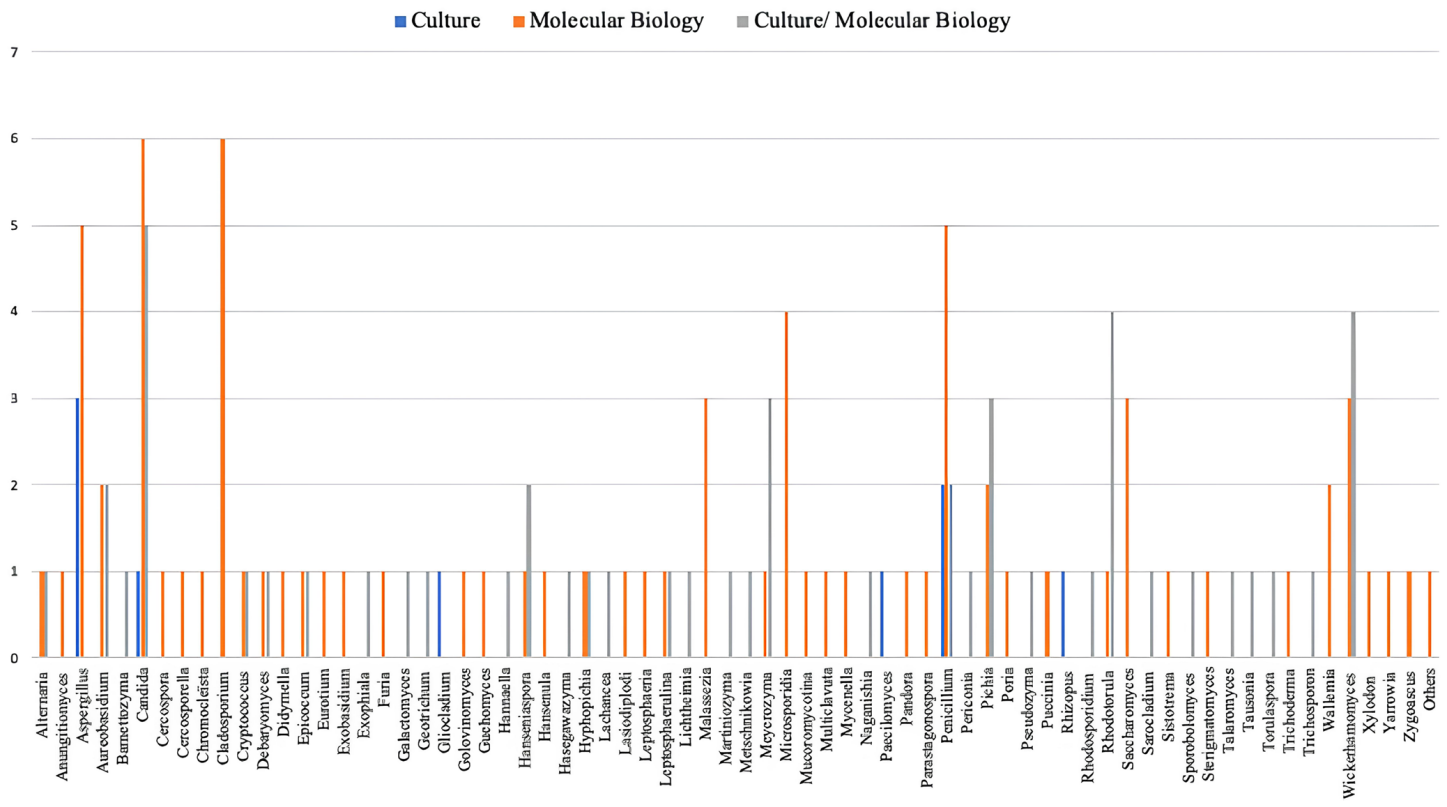
Distribution of fungal genera in *Aedes*, *Anopheles* and *Culex* mosquito microbiota by isolation and identification techniques.

The analyses of fungal genera according to the techniques used for isolation and identification such as culture-dependent techniques involving homogenate culture on agar media, molecular biology techniques on homogenate and molecular biology techniques coupled culture- techniques of homogenate on agar media ( Figure 4).

**Fungal diversity according to culture-dependent technique**

The culture-dependent technique for isolating fungal genera appears to be the least used in studies. Thus, *Aspergillus* and *Penicillium* are the two predominant filamentous fungal genera isolated from the microbiota using the culture technique. In addition, this technique allows the isolation of small proportions of other fungal genera, including 1 yeast-like species such as *Candida* and 2 filamentous species ( *Gliocladium*, *Paecilomyces*, *Rhizopus*) ( Galal *et al.*, 2017; da S Pereira *et al.*, 2009; Tajedin *et al.*, 2009).

**Fungal diversity according to molecular biology techniques coupled with culture**

In numerous studies, it has been possible to isolate several fungal genera using molecular biology techniques combined with culture. Most of the fungal genera isolated are yeast-like (7 in total, including *Candida*, *Rhodotorula*, *Hanseniaspora*, *Meyerozyma*, *Pichia*, *Wickerhamomyces*, and *Hanseniaspora*) and filamentous fungi (including *Aureobasidium* and *Penicillium* ( Angleró-Rodríguez *et al.*, 2016; Bozic *et al.*, 2017; Cappelli *et al.*, 2023; Gusmão *et al.*, 2007; Hegde *et al.*, 2024; Malassigné *et al.*, 2025; Ricci *et al.*, 2011a; Steyn *et al.*, 2016).

As for the other minority isolated genera, they include 19 yeast-like species ( *Barnettozyma*, *Cryptococcus*, *Debaryomyces*, *Galactomyces*, *Geotrichum*, *Hannaella*, *Hasegawazyma*, *Hyphopichia*, *Lachancea*, *Martiniozyma*, *Metschnikowia*, *Naganishia*, *Pseudozyma*, *Rhodosporidium*, *Sporobolomyces*, *Tausonia*, *Torulaspora*, *Trichosporo*n, *Yarrowia*) ( Bozic *et al.*, 2017; Malassigné *et al.*, 2025; Nattoh *et al.*, 2021a; Steyn *et al.*, 2016). In addition to these yeast-like genera, nine filamentous genera composed of molds and phytopathogens including *Alternaria*, *Epicoccum*, *Exophiala*, *Leptosphaerulina*, *Lichtheimia*, *Periconia*, *Sarocladium*, *Talaromyces* and *Trichoderma* are weakly represented ( Angleró-Rodríguez *et al.*, 2017; Nattoh *et al.*, 2021a; Steyn *et al.*, 2016).

**Fungal diversity according to molecular biology techniques**

Given the sensitivity of this technique, it allowed the identification of several other fungal genera in addition to those isolated by the association of molecular biology technique with culture. technique The predominant fungi consist of seven yeast-like genera such as *Candida* ( Bozic *et al.*, 2017; Flores *et al.*, 2022; Gusmão *et al.*, 2010; Hegde *et al.*, 2024; Luis *et al.*, 2019; Ricci *et al.*, 2011a), *Malassezia* ( Chandler *et al.*, 2015; Guégan *et al.*, 2020a; Hegde *et al.*, 2024), *Sacharomyces* ( Bishop-Lilly *et al.*, 2010; Chandler *et al.*, 2015; Hegde *et al.*, 2024) *Wickerhamomyces* ( Muturi *et al.*, 2016; Ricci *et al.*, 2011a; Ricci *et al.*, 2011b), *Hansenula* ( Muturi *et al.*, 2016), Pichia ( Gusmão *et al.*, 2010; Ricci *et al.*, 2011a), *Wallemia* ( Muturi *et al.*, 2016; Ricci *et al.*, 2011a). In addition, an obligate intracellular genus ( *Microsporidia*) and filamentous fungi (5 genera) such as *Cladosporium* ( Chandler *et al.*, 2015; Flores *et al.*, 2022; Guégan *et al.*, 2020a; Luis *et al.*, 2019; Muturi *et al.*, 2016; Thongsripong *et al.*, 2017), *Aspergillus* ( Bishop-Lilly *et al.*, 2010; Chandler *et al.*, 2015; Flores *et al.*, 2022; Luis *et al.*, 2019; Muturi *et al.*, 2016), *Penicillium* ( Bishop-Lilly *et al.*, 2010; Flores *et al.*, 2022; Guégan *et al.*, 2020a; Muturi *et al.*, 2016; Zouache *et al.*, 2022), *Alternari*a ( Muturi *et al.*, 2016), *Aureobasidium* ( Guégan *et al.*, 2020a; Luis *et al.*, 2019) are mostly identified.

The fungal genera weakly identified by this technique include several fungal genera (23), composed of molds such as *Mucoromycotina* ( Flores *et al.*, 2022), *Trichoderma* ( Guégan *et al.*, 2020a), phytopathogens ( *Anungitiomyces*, *Cercospora*, *Cercosporella*, *Didymella*, *Epicoccum*, *Eurotium*, *Exobasidium*, *Golovinomyces*, *Lasiodiplodi*, *Leptosphaeria*, *Leptosphaerulina*, *Parastagonospora*, *Puccinia*, *Sistotrema*, *Xylodon*) ( Chandler *et al.*, 2015; Flores *et al.*, 2022; Guégan *et al.*, 2020a; Muturi *et al.*, 2016), one entomopathogen ( *Furia*, *Pandora*) ( Thongsripong *et al.*, 2017), and other fungi ( *Multiclavuta*, *Mycenella*, *Poria*) ( Chandler *et al.*, 2015; Thongsripong *et al.*, 2017).

In addition, yeast-like genera (9) such as *Cryptococcus*, *Debaryomyces*, *Guehomyces*, *Hanseniaspora*, *Hyphopichia*, *Meyerozyma*, *Rhodotorula*, *Sterigmatomyces*, and *Zygoascus* are also identified in small quantities ( Chandler *et al.*, 2015; Flores *et al.*, 2022; Luis *et al.*, 2019; Muturi *et al.*, 2016).

***Fungal diversity associated with different stages of development of the species Aedes, Anopheles, Culex***

Mosquitoes host a diverse fungal community within their tissues, the composition of which varies according to developmental stage (larvae, pupae, adults) and origin of specimens (field or breeding) (Supplementary file 3. Table III).

**At the larval stages**

The fungal composition of larvae appears to vary depending on the mosquito species, the origin of the mosquito, and the larval habitat.

For example, while larvae of *Ae. aegypti* collected in the field (Lopé village) harbored three (3) phyla ( *Ascomycota*, *Basidiomycota*, *Zycomycota*) ( Zouache *et al.*, 2022), only one phylum ( *Ascomycota*) with the species *Candida parapsilosis* was found at different stages of development (larvae, nymphs) in *Ae. aegypti* from a laboratory breeding program (Camerino) ( Bozic *et al.*, 2017). Several yeasts belonging to two phyla ( *Ascomycota*, *Basidiomycota*), mainly of the genera *Debaryomyces*, *Pichia*, and *Wickerhamomyces*, and a filamentous fungus of the genus *Exophiala* ( *Ascomycota*) were found in *Ae. albopictus* larvae collected in the field (Community garden of Saint Priest) ( Malassigné *et al.*, 2025). In addition, fungal species belonging to four (4) rare fungal phyla ( *Chytridiomycota*, *Glomeromycota*, *Mucoromycota*, *Rozellomycota*) were identified from the carcasses of *Ae. albopictus* larvae collected in the field (Manhattan, KS) ( Tawidian *et al.*, 2021). *Ae. fluviatilis* larvae collected in the field (Municipality of Rio Preto da Eva (Amazonas) hosted a species of the genus *Aspergillus* belonging to the phylum *Ascomycota* ( da S Pereira *et al.*, 2009).

In addition, in *An. gambiae* and *An. stephensi* species reared under laboratory conditions (Camerino), a yeast species, *Candida parapsilosis*, belonging to the *Ascomycota* phylum, was identified at different stages of development (larvae, pupae) ( Bozic *et al.*, 2017). In addition, another yeast species, *Wickerhamomyces anomalus*, was found in *An. gambiae* larvae ( Cappelli *et al.*, 2023), as well as at different stages of development (larvae, pupae) of *An. stephensi* kept in the laboratory (Camerino) ( Ricci *et al.*, 2011a).

Respectively, one filamentous fungal genus ( *Aspergillus*) and three ( *Penicillium*, *Gliocladium*, *Paecilomyces*), all belonging to the phylum *Ascomycota*, were observed in larvae of two mosquito species, *An. argyritarsis* and *An. darlingi*, collected at the same site (Municipality of Rio Preto da Eva (Amazonas) ( da S Pereira *et al.*, 2009).

Like other species, *Cx. quinquefasciatus*, bred in the laboratory (Camerino), also hosted the species *Candida parapsilosis* at different larval stages (larvae, pupae) ( Bozic *et al.*, 2017). In addition to *Candida parapsilosis*, other species belonging to six genera comprising molds (Aspergillus, *Cladosporium*, *Penicilium*, *Mucoromycotina*) and yeasts ( *Debaryomyces*, *Sterigmatomyces*) were isolated from *Cx. quinquefasciatus* larvae kept under laboratory conditions (Argentina) ( Flores *et al.*, 2022). In addition, filamentous fungal species ( *Aspergillus flavus*, *Penicillium notatum*, *Rhizopus stolonifer*) and yeast-like fungal species ( *Candida parapsilosis, Candida pseudolambica*, *Rhodotorula mucilaginosa*, *Rhodosporidium diobovatum*, *Wickerhamomyces anomalus*) were found respectively in larvae kept under laboratory conditions (Egypt) ( Galal *et al.*, 2017) and larvae collected in the field (South Africa) ( Steyn *et al.*, 2016). From the same collection site (South Africa) as the *Cx. pipiens* larvae, the *Cx. theileri* larvae hosted more fungal species belonging to nine genera ( *Candida*, *Cryptococcus*, *Galactomyces*, *Hannaella*, *Meyerozyma*, *Pichia*, *Rhodosporidium*, *Rhodotorula*, *Trichosporon*) ( Steyn *et al.*, 2016).

Finally, fungal species such as *Penicillium georgiense*, *Penicillium notatum*, and *Aspergillus flavus* were identified in *An. gambiae* nymphs and *Cx. pipiens* eggs ( Galal *et al.*, 2017; Nattoh *et al.*, 2021a), highlighting the persistence of fungal species beyond the larval stage.

***In the adults stages***

Adult mosquitoes were found to harbor more diverse mycobiota. The major role of female mosquitoes in pathogens transmission justify the fact that several studies were primarily focused primarily on fungal diversity of these females mosquitoes.

Fungal genera including molds such as *Aspergillus* and *Penicillium* and a yeast such as *Saccharomyces*, all belonging to the phylum *Ascomycota*, have been identified in female *Ae. aegypti* mosquitoes bred in the laboratory ( Bishop-Lilly *et al.*, 2010). As for *Ae. albopictus*, wild females harbored molds (genera *Aspergillus*, *Aureobasidium*, *Cladosporium*), yeasts (genera *Candida*, *Hyphopichia*) belonging to the phyla *Ascomycota* ( Bishop-Lilly *et al.*, 2010), and only yeasts (genera *Candida*, *Lachancea*, *Malassezia*, *Rhodotorula*, *Saccharomyces*) belonging to the phyla ( *Ascomycota*, *Basidiomycota*) ( Hegde *et al.*, 2024). In addition, a yeast-like fungus *Wickerhamomyces anomalus*, belonging to the phylum *Ascomycota*, has been found in male *Ae. albopictus* ( Cappelli *et al.*, 2023).

*Microsporidia* MB, an obligate intracellular parasitic fungus belonging to the phylum *Microsporidiomycota*, has been identified in wild gravid and non-gravid females and in the carcasses of males *An. arabiensis* ( Herren *et al.*, 2020; Nattoh *et al.*, 2021b; Onchuru *et al.*, 2024). In male and female *An. stephensi* mosquitoes from an insectary (Camerino), several yeast-like fungi, including the genera *Candida*, *Pichia*, *Wickerhamomyces*, *Hanseniaspora*, and *Wallemia* belonging to the phyla *Ascomycota* and *Basidiomycota*, were isolated ( Ricci *et al.*, 2011a).

*Culex* mosquito species (particularly *Cx. pipiens*, *Cx. quinquefasciatus*, and *Cx. fucoscephala*) host a wide variety of fungi, including filamentous fungi (molds, phytopathogens) and yeast-like fungi. Several molds (genera *Aspergillu*s, *Cladosporium*), yeasts ( *Guehomyces*, *Malassezia*, *Saccharomyces*), phytopathogens ( *Cercosporella*, *Eurotium*) and other fungi ( *Chromocleïsta*, *Multiclavuta*, *Mycenella*, *Poria*) have been found in wild female *Cx. pipiens* ( Chandler *et al.*, 2015). Fungal genera consisting of a mold ( *Aureobasidium*), yeasts ( *Candida*, *Cryptococcus*, *Pseudozyma*), and a phytopathogen ( *Sarocladium*) were also identified in wild adults of *Cx. pipiens* ( Steyn *et al.*, 2016). When female *Cx. pipiens* were kept under laboratory conditions, molds (genera *Aspergillus*, *Penicillium*, *Rhizopus*) and a yeast ( *Candida*) were found ( Galal *et al.*, 2017). In addition, several yeast-like fungal genera (Candida, *Malassezia*, *Saccharomyces*) ( Hegde *et al.*, 2024), a filamentous genus ( *Cladosporium*), an entomopathogenic genus ( *Furia*) ( Thongsripong *et al.*, 2017), and another genus ( *Pandora*) ( Thongsripong *et al.*, 2017) have been found in wild female *Cx. quinquefasciatus*. In one study, only yeast-like fungal species, including *Candida oleophila*, *Lachancea thermotolerans*, and *Rhodotorula mucilaginosa*, were isolated from adult *Cx. quinquefasciatus* maintained under laboratory conditions ( Hegde *et al.*, 2024).

Finally, in one study, diet appears to influence fungal diversity in male and female *Cx. quinquefasciatus* kept under laboratory conditions ( Flores *et al.*, 2022). After feeding on sugar, males and females harbored several identical fungal genera, including molds ( *Aspergillus*, *Cladosporium*), yeasts ( *Candida*, *Rhodotorula*, *Sterigmatomyces*), and a phytopathogen ( *Didymella*) ( Flores *et al.*, 2022). Fungal genera such as *Penicillium* and *Mucoromycotina* were specifically isolated in sugar-fed males of *Cx. quinquefasciatus* ( Flores *et al.*, 2022). Blood fed females were found blood to harbor mostly molds ( *Anungitiomyces*, *Aspergillus*, *Cladosporium*) and a phytopathogen (genus *Puccinia*) fungi ( Flores *et al.*, 2022). In summary, the molds fungi such as *Aspergillus* and *Cladosporium* are found in both sugar-fed male *Cx*. q *uinquefasciatu*s and blood- or sugar-fed females ( Flores *et al.*, 2022).

**In the digestive organs**

Known for its important role in immunity and metabolism ( Muturi *et al.*, 2016), the gut is the first place where pathogens are transported in infested mosquitoes ( Heu & Gendrin, 2018). It is one of the most studied organs and constitutes an important fungal microhabitat. A yeast-like fungus (genus *Meyerozyma*) and a mold ( *Talaromyces*) have been identified in the intestines ( Bozic *et al.*, 2017) and midguts of wild female *Ae. aegypti* ( Angleró-Rodríguez *et al.*, 2017), respectively *Wickerhamomyces anomalus* ( Ricci *et al.*, 2011b) and *Candida parapsilosis* ( Bozic *et al.*, 2017), two yeast species belonging to the phylum *Ascomycota*, were found in the intestines of adults of *Ae. aegypti* and *Ae. albopictus*, respectively, kept under laboratory breeding conditions. Similarly, this yeast species ( *Wickerhamomyces anomalus*) has been identified in the intestines of wild *Ae. aegypti* and *Ae. albopictus* ( Ricci *et al.*, 2011b). A study showed that newly emerged female *Ae. aegypti* kept in laboratory and fed a diet with sucrose and blood, harbored several fungi species belonging to two genera including *Candida* and *Pichia* isolated from the intestines and intestinal diverticula ( Gusmão *et al.*, 2010). Several fungal genera composed of molds ( *Aureobasidium*, *Cladosporium*, *Penicillium*, *Trichoderma*), a yeast ( *Malassezia*), and phytopathogens ( *Golovinomyces*, *Lasiodiplodia*, *Leptosphaeria*, *Parastagonospora*, *Xylodon*) were identified in the intestines and crops of *Ae. albopictus* mosquitoes kept in the laboratory ( Guégan *et al.*, 2020a). Two species of yeast, *Meyerozyma guilliermondii* and *Sporobolomyces cf. roseus*, belonging to the phyla *Ascomycota* and *Basidiomycota*, respectively, have also been identified in the intestines of wild female *Ae. albopictus* ( Bozic *et al.*, 2017). As for wild females of *Ae. japonicus* and *Ae. triseriatus*, several fungal genera, including yeasts ( *Cryptoccoccus*, *Hanseniaspora*, *Hansenula*, *Meyerozyma*, *Wallemia*, *Wickerhamomyces*, *Yarrowia*), molds ( *Alternaria*, *Aspergillus*, *Cladosporium*, *Penicillium*) and a phytopathogen ( *Leptosphaerulina*) have been identified in the midguts ( Muturi *et al.*, 2016). A proliferation of the species *Meyerozyma caribbica* was observed in the midguts of both *Ae. japonicus* and *Ae. triseriatus* mosquitoes fed on infected blood.

In addition, the yeast species *Wickerhamomyces anomalus* has been identified in the intestines of adult *An. gambiae* and *An. stephensi* maintained in different laboratories ( Ricci *et al.*, 2011b; Ricci *et al.*, 2011b). Similarly, *Candida parapsilosis* has been identified in the intestines of adult *An. gambiae* and *An. stephensi* kept in the laboratory and also in the intestines of wild female *An. gambiae* ( Bozic *et al.*, 2017). Other fungal species belonging to the genera ( *Rhodotorula* and *Meyerozyma*) ( Bozic *et al.*, 2017) and ( *Candida*, *Pichia*, and *Meyerozyma*) ( Bozic *et al.*, 2017; Ricci *et al.*, 2011a) have been detected in the intestines of female *An. gambiae* and *An. stephensi* kept under laboratory conditions ( Bozic *et al.*, 2017; Ricci *et al.*, 2011a). In wild female *An. gambiae*, fungal genera including a yeast ( *Hyphopichia*) and phytopathogens ( *Lichtheimia*, *Lepthosphaerulina*, *Epicoccum*, *Periconia*) have been identified in the intestines ( Nattoh *et al.*, 2021a). *Penicillium chrysogenum*, a species of mold, was also found in the midguts of wild adults of *An. gambiae* and *An. stephensi* ( Angleró-Rodríguez *et al.*, 2016). *Microsporidia* MB, an obligate intracellular fungal species, has been detected in the abdomen of wild females of *An. arabiensis*, *An. gambiae* s.l., and *An. gambiae* s.s. ( Akorli *et al.*, 2024), as well as in the midguts of male *An. arabiensis* kept in the insectarium ( Nattoh *et al.*, 2021b).

A study identified also the yeast *Meyerozyma guilliermondii*, in the intestines of wild female *Cx. quinquefasciatus*, as well as another fungi species including *Candida parapsilosis*, in the intestines of wild adult *Cx. quinquefasciatus* and those kept under laboratory conditions ( Bozic *et al.*, 2017).

**In the reproductive organs**

The analyse showed that mosquitoes harbor several yeast-like fungi species in their reproductive organs.

Two species of yeast, *Candida parapsilosis* and *Wickerhamomyces anomalus*, have been identified in the reproductive organs (male and female) of *Ae. aegypti* and *Ae. albopictus* kept under laboratory conditions ( Bozic *et al.*, 2017; Ricci *et al.*, 2011). In addition, yeasts of the genus *Candida* and *Pichia* were found in the ovaries of newly emerged female *Ae. aegypti* kept in the laboratory ( Gusmão *et al.*, 2010), highlighting possible transmission via the reproductive organs.

*Candida parapsilosis* and *Wickerhamomyces anomalus*, two species of yeast, have been found in the reproductive organs (male and female) of *An. gambiae* and *An. stephensi* kept in various laboratories ( Bozic *et al.*, 2017; Ricci *et al.*, 2011). A yeast-like fungal genus ( *Hyphopichia*) and phytopathogens ( *Leptosphaerulina*, *Lichtheimia*, *Epicoccum*, *Periconia*) have also been isolated in the ovaries of wild female *An. gambiae* ( Nattoh *et al.*, 2021a). In addition, the intracellular fungus Microsporidian MB has also been identified in the gonads of male *An. arabiensis* kept in the insectarium ( Nattoh *et al.*, 2021b).

Finally, the species *Candida parapsilosis* has been identified in the reproductive organs (male and female) of wild *Cx. quinquefasciatus* and those kept under laboratory conditions ( Bozic *et al.*, 2017).

***Vector control potential of mosquito fungal microbiota species***

Certain fungal species associated with mosquito microbiota show significant potential in vector control, either by inhibiting the development of larval stages or by negatively influencing the transmission of pathogens ( Table IV). For example, fungal species in the mosquito microbiota such as *Microsporidia* MB and *Wickerhamomyces anomalus* have demonstrated their effects by inhibiting the transmission of the *Plasmodium* parasite in *An. arabiensis* ( Herren *et al.*, 2020) and by reducing vector competence in *An. stephensi* ( Cappelli *et al.*, 2014; Cappelli *et al.*, 2023; Valzano *et al.*, 2016).

**Table IV.** Potential of fungal species in the microbiota of mosquitoes for vector control.

Fungi species	Life history traits	Impact on vector competence	Pathogen used	Dev stage used	Mosquitoes species	Reference
*Microsporidia MB*	No survival and fecundity effect	Impairs the transmission	*Plasmodium*	Adults	*Anopheles arabiensis*	( Herren *et al.*, 2020)
*Leptosphaerulina* sp	Delayed the development time	-		Larvae	*Anopheles gambiae*	( Nattoh *et al.*, 2021b)
*Wickerhamomyces anomalus*	-	Inhibitory activity on the development of early sporogonic stages	*Plasmodium*	Adults	*Anopheles stephensi*	( Cappelli *et al.*, 2014; Cappelli *et al.*, 2019; Valzano *et al.*, 2016)
*Candida albicans*	Reduced survival rate, inhibits pupation		-	Larvae	*Culex pipiens*	( Steyn *et al.*, 2016)
*Candida glabrata*	Gradual reduction of larvae	**-**	**-**	Larvae	*Culex pipiens*	( Steyn *et al.*, 2016)
*Candida pseudolambica*	Slows larvae growth			Larvae	*Culex pipiens*	( Steyn *et al.*, 2016)
*Martiniozyma asiatica*	Slower development, produces smaller adult	**-**	**-**	Larvae	*Aedes albopictus*	( Malassigné *et al.*, 2025)
*Torulaspora delbrueckii*	Slower development, produces smaller adult	**-**	**-**	Larvae	*Aedes albopictus*	( Malassigné *et al.*, 2025)

- : No information

In addition, a filamentous fungus, *Leptosphaerulina* sp, isolated from the midgut of *An. gambiae* mosquitoes, delayed the development time of larvae when reintroduced by co-feeding from the larval stage except stage 1 (L1). Also, fungal species such as *Candida albicans*, *Candida glabrata*, and *Candida pseudolambica* isolated from *Culex* larvae ( *Cx. pipiens*, *Cx. theileri*) caused a reduction in survival rate followed by inhibition of pupation, a gradual reduction in larvae, and a slowdown in larval growth when reintroduced by co-feeding in *Cx. pipiens* larvae ( Steyn *et al.*, 2016). In addition, other yeast species such as *Martiniozyma asiatica* and *Torulaspora delbrueckii* caused a delay in larval development, resulting in small adults when they were reintroduced through co-feeding in *Ae. albopictus* larvae ( Malassigné *et al.*, 2025).

## Discussion

### General interpretation of the results

This systematic review showed that several studies on *Aedes*, *Anopheles*, and *Culex* mosquitoes microbiota fungal diversity have been published around the world. Most of these studies have been conducted in countries such as Italy, the United States, Brazil, France, Kenya, France, and Thailand. Few studies have been conducted in Argentina, South Asia, India, Vietnam, and a few African countries (South Africa, Burkina Faso, Egypt, Gabon, Ghana, Madagascar). In countries such as the United States, Brazil, European countries (France, Italy), and Kenya, most studies on mosquito microbiota are part of research for alternative and innovative tools for vector control. Research in this field of mosquito microbiota requires a certain medical entomology and microbiology expertise, research founds, and lab facilities that are not common in all countries. These factors could explain why mosquito fungal microbiota remains largely uncharacterized Africa and Asia despite the fact of the burden of vector-borne diseases in these countries.

During this systematic review, fungal genera, more than 41 (with more than 63 species), 19 (with 26 species), and 36 (with 54 species) belonging mainly to the phyla *Ascomycota* followed by *Basidiomycota* were detected in species of the genera *Aedes*, *Anopheles*, and *Culex*, respectively. In addition to the phyla *Ascomycota* and *Basidiomycota*, species of the genus *Anopheles* hosted a fungal genus ( *Microsporidia*) belonging to the phylum ( *Microsporidiomycota*). Species of the genus *Aedes* hosted more fungal genera, followed by species of *Culex* and species of *Anopheles*. This difference in fungal species among these vectors is partly due to their ecology. Although all mosquitoes share many biological traits, the preferred habitats are often specific to the different genera *Aedes*, *Anopheles*, and *Culex*. As cosmopolitan mosquito species, *Aedes* and *Culex* have preferred habitats that can be dark, stagnant water, such as puddles and water containers containing organic matter ( Ferede *et al.*, 2018; Heu & Gendrin, 2018; Kahamba *et al.*, 2020; Krol *et al.*, 2024; Monteiro *et al.*, 2019) that contribute to the proliferation of yeast-like fungi and certain molds. The different species of the genus *Anopheles*, on the other hand, have different aquatic habitats, which may be clear water exposed to sunlight, such as puddles and hoofprints left by animals, for some, and lakes, swamps, and rice fields for others ( Heu & Gendrin, 2018; WHO, 2022), resulting in a low presence of fungi.

Molecular biology-based approaches, whether or not combined with agar culture, and agar culture-based techniques have enabled the identification of different fungal species. The molecular biology-based technique enabled the identification of 46 fungal genera (more than 64 species), followed by 34 fungal genera (57 species) using the molecular biology technique combined with culture, and 6 fungal genera (10 species) using the culture-based technique alone. Due to their sensitivity and specificity, molecular biology-based methods (simple PCR, PCR followed by high-throughput sequencing or metagenomic sequencing) ( Flores *et al.*, 2022; Guégan *et al.*, 2020a; Hegde *et al.*, 2024) offer the best performance in identifying fungal species. This technique allows the identification of non-cultivable species such as *Microsporidia* ( Akorli *et al.*, 2024; Herren *et al.*, 2020; Nattoh *et al.*, 2021b; Onchuru *et al.*, 2024), cultivable species, species present in low quantities in homogenates, and even species sensitive to environmental stress. In addition, molecular biology techniques combined with culture enable the identification of several fungal species, demonstrating that a large proportion of the fungal species in the mosquito microbiota are cultivable. The molecular biology method combined with culture appears to be effective because it ensures the viability of the identified fungal species ( Steyn *et al.*, 2016). Few fungal species have been identified using the culture-dependent identification method alone.

In the larval stages, the *Aedes*, *Anopheles*, and *Culex* species harbored a fairly diverse fungal microbiota with 31 genera comprising 52 species belonging to eight phyla, mainly the phyla *Ascomycota* and *Basidiomycota*. Mostly yeast-like fungi (21 genera) with *Candida*, *Meyerozyma*, *Pichia*, *Rhodotorula*, and *Wicherhamomyces*, followed by molds (8 genera), including *Penicillium* and *Aspergillus. Similarly*, one genus of phytopathogenic fungi ( *Gliocladium*) and one genus of entomopathogenic fungi ( *Paecilomyces*) were associated with the larvae. These fungal communities associated with larval stages often varied between mosquito species and different larval environments (field/laboratory). Several studies have shown variation in fungal communities between different larval environments and between individuals coexisting in the same environment ( Shelomi, 2019; Tawidian *et al.*, 2021). This diversity in the larval stages is thought to be due to the environment of the breeding sites. Female mosquitoes lay their eggs in breeding sites that vary according to the biotope and species ( Fontenille *et al.*, 2017). After hatching, the eggs produce larvae and the mosquito larvae belonging to the *Culicinae* and *Anophelinae* subfamilies are aquatic and acquire part of their microbiota for feeding on organic particles contained in the water of their breeding sites, single-celled organisms (such as bacteria, fungi, and protozoa), and small invertebrates ( Fontenille *et al.*, 2017; Hawkes & Hopkins, 2022; Heu & Gendrin, 2018). These organic particles contribute to the proliferation of fungi in mosquito larvae.

In addition, adults of the *Aedes*, *Anopheles*, and *Culex* species harbored a fungal microbiota that was as diverse in genus (33) belonging to six phyla, predominantly three phyla ( *Ascomycota*, *Basidiomycota*, *Microsporidiomycota*) with fewer species (45) than the larval stages. It appears that adult mosquitoes acquire part of their microbiota during emergence by absorbing water from larval habitats ( Malassigné *et al.*, 2020; Steyn *et al.*, 2016). However, as observed for bacteria, a significant reduction in fungal diversity is observed in newly emerged adults following the process of metamorphosis from nymph to adult ( Malassigné *et al.*, 2020; Steyn *et al.*, 2016). However, the fungal species consisted of yeasts (14 genera), phytopathogens (11 genera), molds (5 genera), entomopathogens (2 genera), and an obligate intracellular fungus that could vary depending on the origin of the mosquitoes (field/laboratory). Several fungal species, predominantly *Candida*, *Aspergillus*, *Cladosporium*, *Penicillium*, and *Rhodotorula*, were isolated from adults (males and females) kept under different breeding conditions in laboratories. The abundance of these fungal species in adults (males and females) could be linked to fungal recolonization after emergence, following reintroduction through feeding habits ( Flores *et al.*, 2022), or through contaminants previously isolated in drinking water ( Novak *et al.*, 2016). Also, as symbionts involved in nutrition in adult male *Ae. albopictus* ( Guégan *et al.*, 2020a), *Aspergillus* and *Cladosporium* molds have been found in both sugar-fed male *Cx. quinquefasciatus* and blood- or sugar-fed females, suggesting a possible involvement in nutrition in adult (male and female) *Cx. quinquefasciatus*. Wild mosquitoes, on the other hand, have a greater fungal diversity than those kept in laboratory conditions. This fungal diversity in the microbiota is thought to be partly due to the nutritional behavior of wild mosquitoes. In addition to being hematophagous at one point in their life cycle, female mosquitoes of the *Aedes*, *Culex*, and *Anopheles* genera in the wild also feed on sugary juices (flower nectar, tree sap, fruit exudates) like male mosquitoes ( Fontenille *et al.*, 2017; Hawkes & Hopkins, 2022; Heu & Gendrin, 2018). These mosquitoes prefer nectars rich in monosaccharides (glucose and fructose) and nectars rich in sucrose ( Guégan *et al.*, 2022). Yeasts produced by the fermentation of sugar in nectars attract mosquitoes ( Luis *et al.*, 2019), which also spread them throughout the environment. It turns out that filamentous fungi such as *Cladosporium* and *Aspergillus* are metabolically active in the digestion of fructose in male *Ae. albopictus*, and *Malassezia* yeast is metabolically active in the digestion of fructose in adult male and female *Ae. albopictus* ( Guégan *et al.*, 2020b). Mosquitoes can also get infected through contact with fungi present in the environment, such as *Microsporidia* ( Nattoh *et al.*, 2021b) and many other fungi.

In addition, digestive organs such as the intestines, crop, intestinal diverticulum, and mosquito species of the genera *Aedes*, *Anopheles*, and *Culex* harbor a diverse fungal community belonging to seven (7) phyla, with the phylum *Ascomycota* being the most prevalent, followed by *Basidiomycota*. These fungal species include yeasts, molds, phytopathogens, and an intracellular fungus. The presence of fungi (yeast, filamentous) in these digestive organs could be linked to nutrition. These organs play important roles in nutrition in general. In mosquitoes, the diverticulum is involved in storing sugar meals such as floral nectar before they slowly pass into the intestine to be digested ( Gusmão *et al.*, 2007). As for the crop, it is involved in the selective storage of sugary solutions (nectar) before they are transferred to the mosquito's intestine ( Calkins *et al.*, 2017; Guégan *et al.*, 2020a), as well as the passage of protein-rich blood meals before they are transferred to the intestine in female mosquitoes. The intestine is involved in the digestion and absorption of sugar, which is used as a regular source of energy for mosquitoes, but also for the active intestinal microbiota, including fungi ( Malassigné *et al.*, 2020).

Furthermore, certain species of fungi (yeasts, molds, phytopathogens) belonging to the phylum *Ascomycota* can colonize the male and female genital organs of certain mosquito species ( *Ae. aegypti*, *Ae. albopictus*, *An. gambiae*, *An. stephensi*, and *Cx. quinquefasciatus*). In addition, an obligate intracellular fungus belonging to the phylum *Microsporidiomycota* has been found in the male and female genital organs of *An. arabiensis* ( Nattoh *et al.*, 2021b; Onchuru *et al.*, 2024). The isolation of certain yeast species ( *Wickerhamomyces anomalus*, *Candida parapsilosis*) in the genital organs suggests a possibility of vertical transmission given their presence at all stages of development of certain mosquito species ( Bozic *et al.*, 2017; Ricci *et al.*, 2011; Ricci *et al.*, 2011). Horizontal sexual transmission between adults is also possible with the *Microsporidia* MB species, given that it is isolated solely in the gonads ( Nattoh *et al.*, 2021b). Other fungi found in the genitals, however, originated from the proliferation of environmental contaminants.

Finally, fungal species constitute a well resorce to support vector control ambition. They are able of inhibiting the development of the larval stages of certain species of *Aedes*, *Anopheles*, and *Culex*, or by inhibiting *Plasmodium* transmission or reducing the vectorial competence.

The inhibition of larval development may be due to competition between certain fungal species and the larvae for nutrients available in the water in the breeding sites, the production of toxins capable of inhibiting the larvae, and pollution of the water in the breeding sites.

As for inhibiting *Plasmodium* transmission or reducing the vector competence of *Anopheles*, one study has shown that the microbiota associated with mosquitoes can influence this by producing toxins and modulating the mosquito's immune system ( Malassigné *et al.*, 2020). Indeed, the reduction in vector competence would be possible thanks to competition between the parasite and fungal species for nutrients available in the intestine or stomach, and the production of toxins that are often broad-spectrum antimicrobials (e.g., *Wickerhamomyces anomalus*) ( Cappelli *et al.*, 2014). In addition, the reduction in vector competence may be due to an imbalance created by fungal species with microorganisms (bacteria and/or viruses and/or fungi) that promote the development of the parasite ( *Plasmodium*), or to the regulation of the immune system of mosquitoes ( *Anopheles*), leading to the activation of the main defense pathways, which reduces the survival and proliferation of the pathogen.

### Limitations of the study

This study gathered and synthesized valuable information on *Aedes*, *Anopheles* and *Culex* mosquitoes fungi microbiota, but this contribution to the field do not exclude some has limitations of the study. First, articles were searched for in two databases (PubMed, Google Scholar) that are accessible free of charge without registration. Therefore, data on the fungal microbiota of species of the genera *Aedes*, *Anopheles*, and *Culex* remain insufficient. Second, for the creation of the geographical map, when the studies did not indicate the mosquito collection sites, we assigned the country of study ( Bozic *et al.*, 2017; Cappelli *et al.*, 2023).This geographical correction may be skewed by the absence of these mosquito species in these countries. Finally, the studies included have methodological limitations, with significant differences in sample collection, microbiological analysis, contamination that may originate from the environment, and the sometimes-small sample size.

In addition, the review process was limited by the exclusion of certain articles when the authors were not accessible. These missing data may introduce a selection bias. However, the exclusion of these articles does not impact the overall conclusion of our review. We selected only articles published in English. We may have omitted recent articles in other languages.

### Prospects

***Implications for vector control practice***

Certain fungal species isolated from mosquito microbiota are able to affect larval stages of *Anopheles*, *Aedes*, and *Culex* mosquitoes and may help inhibit the development of *Plasmodium* pathogens. These fungal species can be used as a complement to chemical control methods, such as entomopathogenic fungi ( *Beauveria*, *Metarhizium*). In addition, the results identified a symbiotic yeast, *Wickerhamomyces anomalus*, an intracellular fungus like *Microsporidia* MB, which can block parasite transmission. Furthermore, given the fact that the fungal microbiota community in larval habitats has an impact on mosquito development, the destruction of organic matter and waste could contribute to reducing mosquito density. A good understanding of the ecological role of fungi in the mosquito microbiota will enable targeted use against specific mosquito species and reduce resistance.

***Implications for public health policy***

The results show that fungal microbiota can be integrated into national vector control programs. In integrated control, fungal symbionts can be used in the same way as insecticides and mosquito nets. However, the use of these symbionts in the environment requires support in the form of biosafety policies, impact assessment experts, and regulatory texts. Given the variation in fungal species depending on mosquito species, geographical locations, and ecological conditions, local regulations must be followed to ensure the proper use of fungal microbiota.

***Implications for future research***

The results obtained in this literature review could inspired future research direction. For example, it could be of interest to identifying all fungi in the mosquito microbiota that can further inhibit the development of larval stages or pathogens, to the interactions between mosquitoes, pathogens, and fungi and the associations that may lead to a greater antivector effect between fungi, bacteria, and viruses in the mosquito microbiota. In addition, the results could be of interest to develop tools for manipulating the fungal microbiota through biotechnology, paratransgenesis, and the introduction of competitive or genetically modified fungi, or to understand the interactions between mosquitoes, their fungal microbiota, and their environment.

## Conclusion

This study provides an overview of the published literature on the *Aedes*, *Anopheles*, and *Culex* mosquitoes fungal microbiota and the prospects for their use as biological control tools against vector-borne diseases. Thus, most studies on the fungal microbiota of mosquitoes of the genera *Aedes*, *Anopheles*, and *Culex* have been conducted in Italy, the United States, Brazil, Kenya, France, and Thailand. Species of the genus *Aedes* harbored more fungal genera, followed by *Culex* and *Anopheles*. These species generally belonged to the phyla *Ascomycota* and *Basidiomycota*, with the phylum *Microsporidiomycota* specific to the genus *Anopheles*. The culture of homogenates on agar media allowed the isolation of certain fungal species, especially filamentous fungi. The cultures of homogenates, coupled or not with molecular biology techniques (PCR followed by high-throughput sequencing), allowed for better results in the identification of fungal species. Mostly yeasts of the genus ( *Candida*, *Meyerozyma*, *Pichia*, *Rhodotorula*, and *Wicherhamomyces*), molds ( *Penicillium*, *Aspergillus*), an entomopathogen ( *Paecilomyces*), and phytopathogens ( *Gliocladium*) were identified in mosquitoes at the larval stage. In the adult stages, yeasts of the genus ( *Candida*, *Rhodotorula*) and molds ( *Cladosporium*, *Penicillium*, *Aspergillus*) were found to be dominant in mosquitoes. Specifically, *Microsporidia* MB were found in some species of the genus *Anopheles*. In the digestive organs and certain genital organs, in addition to yeasts, molds, and phytopathogens found in species of the genus *Aedes* and *Culex*, a specific fungus, *Microsporidia* MB, was found specifically in certain species of *Anopheles*. Transovarian transmission and horizontal sexual transmission are thought to be possible specifically through yeasts ( *Wickerhamomyces anomalus*, *Candida parapsilosis*) and an intracellular fungus ( *Microsporidia* MB). Finally, certain fungal species have been found to infect the larval stages of certain species of *Aedes*, *Anopheles*, and *Culex*, while other fungal species ( *Wickerhamomyces anomalus*, *Microsporidia* MB) have been found to reduce the vector capacity of the *Anopheles* genus.

## Registration and protocol

This study could not be registered in the PROSPERO International Prospective Register of Systematic Reviews.

## Data Availability

**Repository name:** *BioStudies*, S-BSST2250 **Title**: Fungal microbiota diversity in *Aedes*, *Anopheles* and *Culex* and their potential use as biological tools to control vector-borne diseases, a systematic review. **Doi**: 10.6019/S-BSST2250 **Licence:** Creative Commons Zero "No rights reserved" data waiver (CC0) **Aïcha Fatimata Sodré, Doubé Lucien Lamy, Nouhoun Traoré, Abdoulaye Diabaté, Fernand Sankara & Etienne Bilgo*** (2025). Fungal microbiota diversity *in Aedes*, *Anopheles* and *Culex* and their potential use as biological tools to control vector-borne diseases, a systematic review. *BioStudies*, S-BSST2250. Retrieved from https://www.ebi.ac.uk/biostudies/studies/S-BSST2250.
