# Supplementary material for: Fungal microbiota diversity in Aedes, Anopheles and Culex and their potential use as biological tools to control vector-borne diseases, a systematic review
Source: Wellcome Open Res. 2026 Feb 23;11:145. [Version 1] doi: 10.12688/wellcomeopenres.25128.1 (PMC13022562; doi:10.12688/wellcomeopenres.25128.1)
Supplement: Peer review history

# Reviewer report: Reviewer response for version 1

**DOI:** 10.21956/wellcomeopenres.27701.r150446

**Published:** 2026-04-10

**Authors:** Emma Camacho

**Affiliations:** 1 Johns Hopkins University, Baltimore, USA

Summary

 This manuscript presents a timely and relevant systematic review synthesizing current knowledge on fungal microbiota associated with major mosquito genera ( *Aedes, Anopheles,* and *Culex*) and their potential application in vector control strategies. Overall, the work is of high relevance to the field, particularly given the urgent need for sustainable alternatives to insecticide-based vector control. I commend the authors for assembling a comprehensive dataset and for framing fungal microbiota as an underexplored yet promising axis for vector control innovation.

 Major comments:

- A major limitation affecting the reproducibility and transparency of this review is the inability to access the Supplementary Data files, which are referenced multiple times. Dataset from Supplementary, Table III is highly relevant to provide a big picture of fungal diversity particularly depending on the origen of the samples (lab-reared or wild-caught). Please ensure that all Supplementary Files are fully accessible, properly linked, and formatted. **This is necessary for publication.**

Minor comments

Overall

- The manuscript would benefit from professional proof-editing, particularly in the Introduction, where grammatical errors, awkward phrasing and repetitions reduce clarity and readability. Some examples below:

*“When toking this blood meal biting infected human or animal hosts, female mosquitoes ingests”*

*“Among these pathogens transmitted by mosquitoes malaria protozoa,lymphatic filariasis (Malassigne et al., 2020; WHO, 2022) an arboviruses”*

In the introduction

 “These pathogens are transported into the insect intestines where…” **clarify this statement to include microfilariae development.**

 “ *Bacterial such as R. felis* **….” R. felis responsible for? Dieme et al refers to experimental infection of A. gambiae mosquitoes with the R. felis and JN et al. is a review demonstrating inclusive evidence that mosquitoes can be true vector for F. tularensis. Please clarify that both studies cited here refer to "transmission potential" not real transmission.**

 “and fungi such as Candida parasilopsis…” **As for previous cited work, work cited here do not report transmission of C. parasilopsis to humans. Please edit.**

 “Unfortunately, the excessive use of insecticides agents raised chemical pollution of the environment and insect resistance ti several molecules (Batson *et al., *2021; Chala & Hamde, 2021; Dahmana & Mediannikov, 2020 **)” 1) Perhaps, you may want to refer to agrochemicals instead of just insecticides. 2) None of these studies explicitly identifies or experimentally test agrochemical molecules to which insects have developed resistance. I am not sure of what is the message that wants to be convey. Please reword this sentence.**

In methods

- “Studies tolking…”

In results

- What “weakly identified” by molecular biology techniques refers to?

In limitations

- Maping of mosquito collection should not be a limitation. The information regarding collection sites for the two articles mentioned can be easily address by contacting the corresponding authors.

Are the rationale for, and objectives of, the Systematic Review clearly stated?

Yes

Is the statistical analysis and its interpretation appropriate?

Not applicable

If this is a Living Systematic Review, is the ‘living’ method appropriate and is the search schedule clearly defined and justified? (‘Living Systematic Review’ or a variation of this term should be included in the title.)

Not applicable

Are sufficient details of the methods and analysis provided to allow replication by others?

No

Are the conclusions drawn adequately supported by the results presented in the review?

Partly

Reviewer Expertise:

Melanin, Fungal biology, Malaria, Mosquito Microbiota

I confirm that I have read this submission and believe that I have an appropriate level of expertise to confirm that it is of an acceptable scientific standard, however I have significant reservations, as outlined above.

# Reviewer report: Reviewer response for version 1

**DOI:** 10.21956/wellcomeopenres.27701.r149916

**Published:** 2026-04-01

**Authors:** Daniel W Pérez-Ramos

**Affiliations:** 1 University of Florida, Florida, USA

The review goal is to narrow the knowledge gap in understanding the mosquito mycobiota or mosquito-associated fungi, and their impact on mosquito biology and pathogen transmission, to propose them as tool for mosquito control. The authors performed a PRISMA 2020 guideline complaint systemic assessment on scientific literature found in two search databases, Google Scholar and PubMed. The cured data from 32 selected papers showed interesting patterns of fungi association with mosquitoes with regards of species, type of tissue, laboratory-reared or field-collected conditions, and locations around the world. However, many aspects of this review need to be re-evaluated.

**Major comments:**

 The introduction section offers information that explains aspect of why mosquitoes are relevant to public health. However, when introducing the mosquito microbiota, and in particular, the mosquito mycobiota, the amount of information is not even or is lacking. The authors would benefit from expanding the content and delving into the topic of mosquito microbiomes, and the many implications it has in mosquito biology while evening the narrative content to introduce the mosquito mycobiome, which is the most relevant topic in the review. The information about public health and insecticide resistance should be just a prelude to the microbiome subject and not take up a big part of the content.

 The result section has too much information to the point that the reader can get discouraged from keeping focus. The order of information is not equal per section, and it is hard to keep track on which mosquitoes or which fungi. The in-text result should not repeat the information that is presented in figures. The authors would benefit to concise this section and expand on the data by creating an informative organized table. Figures 2-4 do not properly reflect good visualization on the takeaway message for the following reasons:

- Figure 2: The information in the map can be a table. The scale used on the map is not correct. For example, the distance between east to west in US is 4,500 km and the scale ends in 200km. If the map is the choice from the authors, I suggest deleting the scale as it is not relevant to the highlighted message. I also recommend modifying the legend so that it is inserted on top of the image on an empty space, to avoid stretching on the figure.
- Figure 3: The figure is not readable, and not color-blind friendly. Both axes are not labelled, and the y-axis is wrong as there should not be a 0.5 parameter. The number of variables is far too much for a bar graph. This figure would be beneficial if turned into a table where the variables: associated fungi, mosquito species, and numbers of references can be attributed to three columns in a more organized matter.
- Figure 4: The figure is not readable, and the same recommendation from Figure 3 is applied here as well.

The Discussion section is a repeat of the result section, but more informative. In here the authors expand a little about molecular biology but missed their opportunity to expand more into the topic. Molecular biology is a field of science that uses many techniques not just "a technique", and the authors missed their opportunity to expand on the knowledge of the techniques used in the 32 studies (PCR, qPCR, high-throughput sequencing, metagenomic sequencing). What is the importance of these techniques? What are their limitations in assessing the fungal kingdom? Which of the 32 studies used x, y, z molecular techniques? And why complementing it with microbiological culturing was better in some of the cases? The authors also missed the chance to expand on culturing techniques used by the 32 studies.

 In the Prospect section, the authors missed a few important topics to expand. When mentioning the prospect for vector control practice, the key message on entomopathogenic fungi was explained to a minimum, only two examples were used to explain importance. But what does this mean for vector control and how can it be managed? That part was not explored. Likewise, if using the mycobiome as a tool of vector control and with this, explore those with entomopathogenic phenotypes, how would this impact non-target species if deployed in field conditions? This is a good area to expand prospect and limitations, potentially using other pest insects as examples, specifically since fungi like Metarhizium has been referenced.

 The conclusion was not proper and did not address the big picture. It summarized the content in one paragraph but does not convince the reader why understanding the mosquito mycobiota is beneficial to vector control.

 The authors should explain why the review was not registered in PROSPERO as part of their limitations.

 The manuscript needs a revision from a professional English editor to correct grammar and inconsistencies.

**Minor comments:**

- When introducing “Culicidae family”, make sure that is stated only once. The way the text is written makes the allusion that there is more than one family when brought up for a second time.
- Please avoid vague language like saying several or certain and not expanding on which (i.e. “[…] insecticide resistance ti [ *sic*] several molecules”). Similarly, please avoid overgeneralizations, like stating that all pathogens follow the same infection pathways of tissues in the mosquito.
- Correct typographic errors: toking (talking), et (and), ti (to), France (repeated twice in Discussion), or sudden break of paragraphs (page 9).
- Avoid using wording such as high diversity or low/poor diversity when diversity measures are not performed by the authors. If the authors want to highlight a diversity assessment from a study, clearly state that the cited study found x fungi to be at a lower diversity but avoid the use of this information to imply it will be similar for all systems or misinterpreting that it will be so for a particular mosquito species. Mosquito microbiome is highly variable, even by individuals of the same species.
- *Anopheles gambiae s.l (sensu lato)* and *Anopheles gambiae s.s. (sensu stricto)* are taxonomic terms to include a broad or narrow group of a complex. The inclusion of a third term in the data set: *Anopheles gambiae,* does not make sense. Please review or clarify it use.
- When presenting the mosquito species, i.e. “ *Culex quinquefasciatus* ( *Cx. quinquefasciatus*)” it is not necessary to write the species twice and their genus abbreviation in parentheses. Once a species has been mentioned fully for the first time, you may continue by using the genus abbreviation later in the text. This will save the authors on character count and better readability.
- The term “mycobiota” was used once in the text without proper definition of what this is.
- Mosquitoes are holometabolous, there is no nymphal stage. Life cycle is eggs -> larvae -> pupae -> adult. Please avoid the use of the word “nymph”.
- Keep consistency on terms (i.e. insectarium vs laboratory)

Are the rationale for, and objectives of, the Systematic Review clearly stated?

Yes

Is the statistical analysis and its interpretation appropriate?

Not applicable

If this is a Living Systematic Review, is the ‘living’ method appropriate and is the search schedule clearly defined and justified? (‘Living Systematic Review’ or a variation of this term should be included in the title.)

Not applicable

Are sufficient details of the methods and analysis provided to allow replication by others?

Yes

Are the conclusions drawn adequately supported by the results presented in the review?

No

Reviewer Expertise:

medical entomology, mosquito microbiota, mosquito mycobiota, metabolomics, population genomics

I confirm that I have read this submission and believe that I have an appropriate level of expertise to state that I do not consider it to be of an acceptable scientific standard, for reasons outlined above.

# Reviewer report: Reviewer response for version 1

**DOI:** 10.21956/wellcomeopenres.27701.r150444

**Published:** 2026-03-26

**Authors:** Zhen Zou

**Affiliations:** 1 Chinese Academy of Sciences, Beijing, China

The authors systematically summarize current knowledge on the fungal microbiota associated with three major mosquito groups, *Aedes*, *Anopheles*, and *Culex*. Their methodology involved a structured search of PubMed and Google Scholar, two of most used databases. It yields 32 relevant studies from a pool of 7,458. The review includes the fungal diversity across key variables such as geography, mosquito developmental stage, and specific tissue. It identifies Ascomycota, Basidiomycota, and Microsporidiomycota as the predominant phyla. Critically, the review highlights the functional significance of this mycobiota, distinguishing between entomopathogenic fungi that inhibit larval development and commensal/symbiotic fungi like *Wickerhamomyces anomalus* and *Microsporidia* MB that can reduce the vector competence of adults. The authors adhere to PRISMA guidelines, indicating robust methodological quality and rigorous analysis. It offers a valuable resource for entomologists and vector biologists.

 I didn’t find any big problems. It will be better if the authors could incorporate a section or paragraph that synthesizes the known molecular mechanisms by which these fungi exert their effects. Otherwise, the reading is boring. For example, what are the specific secondary metabolites or immune effector identified in these fungi?

Are the rationale for, and objectives of, the Systematic Review clearly stated?

Yes

Is the statistical analysis and its interpretation appropriate?

Not applicable

If this is a Living Systematic Review, is the ‘living’ method appropriate and is the search schedule clearly defined and justified? (‘Living Systematic Review’ or a variation of this term should be included in the title.)

Not applicable

Are sufficient details of the methods and analysis provided to allow replication by others?

Yes

Are the conclusions drawn adequately supported by the results presented in the review?

Partly

Reviewer Expertise:

vector biology, insect molecular biology, insect genomics

I confirm that I have read this submission and believe that I have an appropriate level of expertise to confirm that it is of an acceptable scientific standard, however I have significant reservations, as outlined above.

# Reviewer report: Reviewer response for version 1

**DOI:** 10.21956/wellcomeopenres.27701.r150438

**Published:** 2026-03-18

**Authors:** Holly Nichols

**Affiliations:** 1 University of Wisconsin-Madison, Madison, USA

Summary

 This systematic review provides a detailed picture of the known diversity and distribution of mosquito-associated fungi. The content and message of the manuscript are timely. While the diversity of mosquito-associated bacteria is well studied, fungal diversity remains relatively understudied. This review provides a comprehensive overview of recent advances in detecting fungal microbiota across host species, life stage, and body site. It also highlights fungal taxa with pathogen-blocking or vector control potential. With a few minor changes, this review will be a much-needed addition to the literature.

 Major Comments:

- Overall, I am skeptical of any definitive comparisons made about the relative fungal diversity of mosquito species due to the methodological variation in the cited papers. It is highly valuable to note which fungal taxa have been detected in various vector species, but the age of the studies cited and the different methods (culture-based collection and identification versus metabarcoding for identification) make it impossible to make claims about which mosquito species have a more diverse microbiota. For example – it is unclear from current evidence whether *Ae. fluviatilis* has a “weak” microbiota – there is only is a single study from a 2009, and it was based on cultivation of fungi which inherently would capture a lower diversity of fungi due to the cultivation bottleneck. Similarly the conclusions for *Ae. japonicus* and *Ae. triseriatus* are from a single paper using metabarcoding/amplicon sequencing. Databases for fungal identification have improved since that study in 2016, and further studies might likely uncover a greater diversity of fungi in species if the study were repeated today. This is something the authors later acknowledge by separating out taxa identified with culture vs molecular biology in Figure 4, but this lens should also be applied to the results sections titled “Fungal diversity associated with the microbiota of (Aedes/Anopheles/Culex) species”. It is also unclear from this part of the manuscript which of these studies used lab- versus field-collected mosquitoes, which undoubtedly influences diversity by species.

Minor comments

- Transmission of bacterial and fungal pathogens are presented as equally verified and dangerous as the more well-established pathogens vectored by mosquitoes (plasmodium, dengue virus, etc…). While it is important to highlight the studies beginning to show potential for mosquitoes to vector these bacterial pathogens and disseminate fungal pathogens, it should be clarified that this is not currently as well-defined as other pathogens, especially for yeasts. The study cited highlights the dispersal potential rather than vectorial capacity for yeasts.
- I would like to commend how thorough the life-stage and tissue-specific portions of the Results section are in mentioning which fungi are detected in lab- versus field- collected mosquitoes, and for including the feeding status where appropriate.
- Language in Table IV is slightly unclear – what does “gradual reduction of larvae” mean and how is it distinguished from other entries that produce “slower larval growth” or “slower development”?  Is this describing larval mortality?
- This sentence: “…fungal genera, more than 41 (with more than 63 species), 19 (with 26 species), and 36 (with 54 species) belonging mainly to the phyla Ascomycota followed by Basidiomycota were detected in species of the genera Aedes, Anopheles, and Culex, respectively.” Might be rewritten for clarity as:
  - “During this systematic review, 41 fungal genera (over 63 species) were detected in Aedes, 19 fungal genera (26 species) were found in *Anopheles*, and 36 fungal genera (54 species) were detected in Culex mosquitoes. “
- In the Discussion section, it would be beneficial to mention how various methodological differences  would affect the big conclusion made about diversity in Aedes vs Culex vs Anopheles – were the techniques (culture-based versus molecular detection) used equally across these mosquito genera? Were the underlying studies collected from similar time periods, since sequencing technology and identification databases have significantly advanced in the study window (2005 – 2025)? Were any of these vector genera biased towards lab- versus field-collected mosquitoes?   
- I think it should be reiterated in the Discussion/Conclusion section how there is so much more to learn on this topic – only a small subset of the fungal genera identified here have been tested for pathogen-blocking activity, and there are doubtless many more mosquito-associated fungi yet to be discovered. This article could prove a comprehensive guide for targeting isolation and subsequent testing of taxa commonly detected  in sequencing studies across vector species for a broader impact. Alternatively, this work could help highlight taxa that are specific to just one vector species in case a narrower vector control approach is desired.
- The manuscript is in need of copyediting for language and clarity, especially in the Introduction, Discussion, and Conclusion sections. A few specific examples are noted below:
  - “…source of protein for **their eggs maturation**” for the maturation of their eggs
  - Toking>> taking
  - “to finally **being** able to be transmitted to a new host during the next blood-feeding” >> be
  - “ **an** arboviruses including the one of dengue fever, yellow fever, chikungunya, Japanese encephalitis and West Nile fever” >> "an" to "and", remove phrase "one of"
  - Arbovirosis > arboviruses
  - Tolking > talking
  - Analyse >> analysis
  - Research founds >> research funds

Are the rationale for, and objectives of, the Systematic Review clearly stated?

Yes

Is the statistical analysis and its interpretation appropriate?

Not applicable

If this is a Living Systematic Review, is the ‘living’ method appropriate and is the search schedule clearly defined and justified? (‘Living Systematic Review’ or a variation of this term should be included in the title.)

Not applicable

Are sufficient details of the methods and analysis provided to allow replication by others?

Yes

Are the conclusions drawn adequately supported by the results presented in the review?

No

Reviewer Expertise:

mosquito microbiota, bacteriology, amplicon sequencing and bacterial genomics

I confirm that I have read this submission and believe that I have an appropriate level of expertise to confirm that it is of an acceptable scientific standard, however I have significant reservations, as outlined above.

# Reviewer report: Reviewer response for version 1

**DOI:** 10.21956/wellcomeopenres.27701.r149045

**Published:** 2026-03-11

**Authors:** Irene Ricci

**Affiliations:** 1 University of Camerino, Camerino, Italy

**Reviewer Report**

**Summary**

 This manuscript presents a systematic review on fungal microbiota diversity in *Aedes*, *Anopheles*, and *Culex* mosquitoes and discusses their potential applications in vector control. The topic is relevant and timely, particularly in the context of insecticide resistance and the search for innovative biological strategies. The review is comprehensive and supported by supplementary materials detailing PRISMA compliance, risk of bias assessment, and study characteristics.

 The supplementary materials adequately support the methodological transparency of the review.

 Overall, the manuscript is suitable for publication after minor revisions.

**Minor comments**

**1. Introduction**

**“When taking this blood…”**

 The description of pathogen development within mosquitoes appears conceptually overgeneralized. While *Plasmodium* parasites and many arboviruses follow a midgut–hemocoel–salivary gland pathway, this is not universally applicable to all mosquito-borne pathogens. For example, filarial nematodes (causative agents of lymphatic filariasis) undergo development in thoracic muscles before migrating to the mouthparts, rather than following the classical salivary gland invasion route.

 In addition, the taxonomy could be clarified. “Lymphatic filariasis” refers to the disease rather than the pathogen; it would be more accurate to refer to filarial nematodes (e.g., *Wuchereria bancrofti*). A clearer distinction between protozoa ( *Plasmodium* spp.), nematodes (filariae), and arboviruses would improve biological precision.

 Please consider revising this paragraph to reflect pathogen-specific developmental pathways within the mosquito vector. The paragraph could be revised as follows:

 “When taking a blood meal from an infected human or animal host, female mosquitoes ingest potential pathogens (Batson et al., 2021; Fontenille et al., 2017). Depending on the pathogen, development within the mosquito may involve infection of the midgut epithelium, dissemination through the hemocoel, and eventual invasion of the salivary glands before transmission to a new host during subsequent blood feeding (Duvallet et al., 2018; Romoli & Gendrin, 2018). Mosquito-borne pathogens include protozoa such as *Plasmodium* spp. (the causative agents of malaria), filarial nematodes responsible for lymphatic filariasis (Malassigné et al., 2020; WHO, 2022), and arboviruses such as those causing dengue, yellow fever, chikungunya, Japanese encephalitis, and West Nile fever (Gao et al., 2020; Malassigné et al., 2020).”

 Minor typographical corrections in the same section include:

 “toking” → *taking*; “ingests” → *ingest*; “malaria protozoa” → *Plasmodium* spp.; “lymphatic filariasis” → filarial nematodes; “an arboviruses” → arboviruses; “bloodfeeding” → blood feeding.

**“Bacteria such as Rickettsia felis…”**

 This paragraph contains grammatical inconsistencies and incomplete sentences. In addition, the wording regarding microbial transmission by mosquitoes (particularly *Candida parapsilosis*) would benefit from clarification to avoid overstatement of vectorial transmission.

 Specifically, the study by Bozic et al. (2017) reports that mosquitoes can harbour yeasts of clinical significance and potentially contribute to their environmental dissemination; however, it does not demonstrate classical biological vector transmission of *Candida parapsilosis*. It may therefore be preferable to use terms such as “carriage,” “association,” or “detection” rather than “transmission,” in order to remain fully aligned with the cited evidence.

 Please revise for grammatical accuracy and conceptual precision. The paragraph could be revised as follows:

 “Mosquitoes are also associated with the transmission or carriage of other microorganisms. Bacteria such as *Rickettsia felis* and *Francisella tularensis* have been detected in mosquitoes, and fungal species such as *Candida parapsilosis* have been reported in association with *Anopheles*, *Aedes*, and *Culex* mosquitoes (Bozic et al., 2017). Vector control constitutes a key component in combating vector-borne diseases. For a long time, insecticides have been the principal tool of this strategy (WHO, 2022). However, their excessive use has led to environmental pollution and the development of resistance to multiple insecticidal compounds (Batson et al., 2021; Chala & Hamde, 2021; Dahmana & Mediannikov, 2020).”

**A careful language revision throughout the manuscript is recommended to improve readability and precision.**

**2. Protocol registration**

 Please briefly clarify the reason why the review was not registered (e.g., PROSPERO eligibility constraints).

**3. Language revision**

 The manuscript would benefit from professional English editing to correct grammatical and typographical errors.

**Recommendation**

 Approve with minor revisions.

Are the rationale for, and objectives of, the Systematic Review clearly stated?

Yes

Is the statistical analysis and its interpretation appropriate?

Not applicable

If this is a Living Systematic Review, is the ‘living’ method appropriate and is the search schedule clearly defined and justified? (‘Living Systematic Review’ or a variation of this term should be included in the title.)

Not applicable

Are sufficient details of the methods and analysis provided to allow replication by others?

Yes

Are the conclusions drawn adequately supported by the results presented in the review?

Yes

Reviewer Expertise:

Parasitology, mosquito-borne diseases

I confirm that I have read this submission and believe that I have an appropriate level of expertise to confirm that it is of an acceptable scientific standard.
